# Bioactive peptides derived from *Radix Angelicae sinensis* inhibit ferroptosis in HT22 cells through direct Keap1–Nrf2 PPI inhibition†

**DOI:** 10.1039/d3ra04057g

**Published:** 2023-07-24

**Authors:** Ban Chen, Xiaojian Ouyang, Chunfeng Cheng, Dongfeng Chen, Jiangtao Su, Yuchen Hu, Xican Li

**Affiliations:** a Key Laboratory of Fermentation Engineering (Ministry of Education), Cooperative Innovation Center of Industrial Fermentation (Ministry of Education & Hubei Province), Hubei University of Technology Wuhan 430068 China; b IncreasePharm Hengqin Inst. Co., Ltd Zhuhai 519000 China oyxiaojian55@163.com; c Shenzhen Hospital of Integrated Traditional Chinese and Western Medicine Shenzhen 518000 China; d School of Basic Medical Science, Guangzhou University of Chinese Medicine Guangzhou 510000 China; e School of Chinese Herbal Medicine, Guangzhou University of Chinese Medicine Guangzhou 510000 China lixican@126.com

## Abstract

The development of natural peptides as direct Kelch-like ECH-associated protein 1 (Keap1)–nuclear factor erythroid2-related factor 2 (Nrf2) protein–protein interaction (PPI) inhibitors for antioxidant and anti-ferroptotic purposes has attracted increasing interest from chemists. *Radix Angelicae sinensis* (RAS) is a widely used traditional Chinese medicine with antioxidant capability. However, few studies have screened Keap1–Nrf2 PPI inhibitory RAS peptides (RASPs). This study optimized the extraction and hydrolysis protocols of RAS protein using response surface methodology coupled with Box–Behnken design. The molecular weight distribution of the prepared hydrolysates was analysed to obtain active fractions. Subsequently, ultra-performance liquid chromatography coupled with electrospray ionization quadrupole time-of-flight tandem mass spectrometry was employed to identify RASPs. Various *in vitro* and *in silico* assays were conducted to evaluate the antioxidant and anti-ferroptotic effects of RASPs. The results revealed that at least 50 RASPs could be obtained through the optimized protocols. RASPs containing active residues effectively scavenged 2,2-diphenyl-1-picrylhydrazyl radical and 2,2′-azinobis(3-ethylbenzothiazoline)-6-sulfonic acid radical cation. They also showed cytoprotective effect against erastin-induced ferroptosis in HT22 cells, which was characterized by the activation of Nrf2 and weakened under the incubation of an Nrf2 inhibitor. Moreover, RASPs could bind to Keap1 and then dissociate Nrf2 in molecular dynamics simulations. In conclusion, RASPs exhibit antioxidant activity through hydrogen atom transfer and electron transfer mechanisms. Importantly, they also inhibit ferroptosis by directly inhibiting Keap1–Nrf2 PPI.

## Abstract 2

Antioxidant peptides derived from *Radix Angelicae sinensis* can inhibit ferroptosis by directly inhibiting Keap1–Nrf2 PPI.

## Graphical abstract

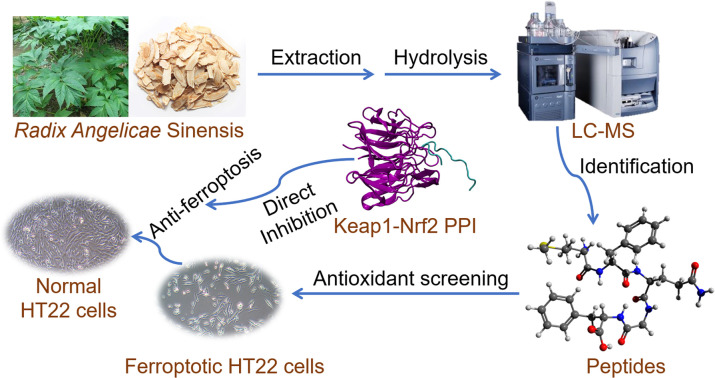

## 1. Introduction

Ferroptosis is a newly identified form of regulated cell death induced by oxidative stress.^1^ Enhancing endogenous antioxidant capacity has great potential for anti-ferroptosis, which is expected to protect normal cells for the treatment of various diseases, such as cancer, Parkinson's disease, and Alzheimer's disease.^2–5^

The endogenous antioxidant capacity can be enhanced through the activation of nuclear factor erythroid2-related factor 2 (Nrf2), a master antioxidant transcription factor negatively regulated by Kelch-like ECH-associated protein 1 (Keap1).^6^ Inhibition of Keap1–Nrf2 protein–protein interaction (PPI) is an effective antioxidant or anti-ferroptotic approach.

Over the past few years, scientists have synthesized a series of Keap1–Nrf2 PPI inhibitors, such as ferrostatin-1 (Fer-1), liproxstatin-1, and entacapone.^7–9^ Most synthetic Keap1–Nrf2 PPI inhibitors contain a sulfonamide or aromatic amine subunit, which has been proven to cause adverse effects such as allergic reactions, digestive symptoms, and liver damage.^10^ Moreover, many synthetic inhibitors covalently bind to Keap1, leading to a conformational change in Keap1. This indirect inhibition is unreversible, resulting in the persistent Nrf2 activity and the risk of cellular carcinogenesis.^11^ It is now generally accepted that a reasonable Keap1–Nrf2 PPI inhibitor should bind Keap1 competitively; in other words, the inhibitor should bind noncovalently to Keap1, which is termed direct inhibition.^11^

To search for direct Keap1–Nrf2 PPI inhibitors, chemists have shifted their focus to natural products. Notably, bioactive peptides originated from plant proteins have captured worldwide attention due to their advantages of environmental protection, sustainability, low cost, and low toxicity.^12–15^ So far, a large number of Keap1–Nrf2 PPI inhibitory peptides have been isolated from plant proteins. For instance, peptides from *Acaudina molpadioides*, cottonseed, and broken rice, exert great antioxidant activity *in vitro* and *in vivo* through Keap1–Nrf2 PPI inhibition.^16–18^

However, most reported Keap1–Nrf2 PPI inhibitory peptides are derived from food proteins for antioxidant investigation, and there are few studies on the anti-ferroptotic effect of Keap1–Nrf2 PPI inhibitory peptides from medicinal plants. In China, *Radix Angelicae sinensis* (RAS) is one of the most common medicinal plants.^19^ It is the dried root of *Angelica sinensis* (Oliv.) Diels and well known as *Danggui* in traditional Chinese medicine.^20^ Recent evidence indicates that RAS peptides (RASPs) can delay the aging process in *Caenorhabditis elegans* based on antioxidant effect,^19^ implying that RASPs may have the potential to inhibit ferroptosis through Keap1–Nrf2 PPI inhibition.

Hence, the present study optimized the extraction and hydrolysis processes of RAS protein using response surface methodology (RSM) coupled with Box–Behnken design (BBD). The molecular weight (MW) distribution of the prepared hydrolysates was further analysed to obtain active fractions. Ultra-performance liquid chromatography coupled with electrospray ionization quadrupole time-of-flight tandem mass spectrometry (UHPLC-ESI-Q-TOF-MS/MS) technology was employed to identify RASPs. Eventually, ten RASP standards were screened and synthesized to investigate their antioxidant and anti-ferroptotic effects based on Keap1–Nrf2 PPI inhibition using various *in vitro* and *in silico* approaches.

## 2. Experimental

### 2.1 Materials and methods

RAS (lot number: 200901181, produced in Gansu Province of China) was purchased from Kangmei Pharmaceutical Co., Ltd (Puning, China). Mouse hippocampal neuronal HT22 cells were purchased from American Type Culture Collection (Rockville, MD, USA). Twenty common amino acids (purity > 95%), including l-alanine (Ala, A), l-arginine (Arg, R), l-asparagine (Asn, N), l-aspartic acid (Asp, D), l-cysteine (Cys, C), l-glutamine (Gln, Q), l-glutamic acid (Glu, E), glycine (Gly, G), l-histidine (His, H), l-isoleucine (Ile, I), l-leucine (Leu, L), l-lysine (Lys, K), l-methionine (Met, M), l-phenylalanine (Phe, F), l-proline (Pro, P), l-serine (Ser, S), l-threonine (Thr, T), l-tryptophan (Trp, W), l-tyrosine (Tyr, Y), and l-valine (Val, V), were purchased from J&K Scientific (Beijing, China). Ten RASPs (purity > 95%), including MFQGF, FQGF, FLLP, VLPQL, FVTP, LLGY, LYN, LAY, TVTY, and VTGGSYG, were synthesized by BankPeptide Inc. (Hefei, China).

Alkaline protease (200 000 U g^−1^), acid protease (50 000 U g^−1^), neutrase (50 000 U g^−1^), pepsin (250 000 U g^−1^), papain (200 000 U g^−1^), trypsin (2.5 million U g^−1^), bovine serum albumin (BSA), and Coomassie brilliant blue G-250 were purchased from Solarbio Science & Technology Co., Ltd (Beijing, China). Glutathione (GSH), Dulbecco's modified Eagle's medium (DMEM), and fetal bovine serum (FBS) were purchased from Gibco (Waltham, USA). Cell counting kit-8 (CCK-8) was purchased from ApexBio Technology (Boston, USA), C11-BODIPY probe was purchased from Molecular Probes (Shanghai, China), erastin was purchased from MedChemExpress (Shanghai, China); Fer-1 was purchased from Glpbio (Shanghai, China), 2,2-diphenyl-1-picrylhydrazyl radical (DPPH˙) was purchased from Sigma-Aldrich (Shanghai China), and 2,2′-azino-bis(3-ethylbenzo-thiazoline-6-sulfonic acid) diammonium salt ((NH_4_)_2_ABTS) was purchased from Amresco (Solon, USA). Cell lysis solution, protease inhibitors, bicinchonininc acid (BCA) protein assay kit, and 4′,6-diamidino-2-phenylindole (DAPI) were purchased from Beyotime Biotechnology (Shanghai, China).

Other main analytically pure reagents included petroleum ether (60–90 °C), CuSO_4_, K_2_SO_4_, H_2_SO_4_, HCl, NaOH, H_3_BO_3_, triethylamine, *n*-hexane, CH_3_COONa·3H_2_O, and CH_3_COOH, all of which were purchased from Zhiyuan Chemical Reagent Co., Ltd (Tianjin, China). Some HPLC grade reagents, such as methanol, ethanol, and formic acid, were purchased from Merck KGaA (Darmstadt, Germany).

### 2.2 Optimization of the extraction process of RAS protein

RAS was dried in an oven at 60 °C, followed by crushing through an 80-mesh sieve to obtain RAS powder. The fat of the prepared powder was repeatedly extracted by petroleum ether at room temperature (25 °C) under continuous stirring until the solution exhibited a colorless appearance from its original yellow hue. The solvent was decanted after each extraction, and the residual powder was completely air-dried at 60 °C.

The crude protein content of the defatted RAS powder was determined using the Kjeldahl method, followed by protein extraction using the alkali extraction and acid precipitation method.^21^ Briefly, RAS powder was suspended in distilled water at a certain concentration, and the pH value of the solution was adjusted using NaOH (1 M). The resulting mixture was centrifuged at 3500*g* for 20 min at room temperature (25 °C), and the supernatant was separated from the insoluble residue. The protein content of the collected supernatant was determined using the Bradford assay.^22^ In brief, 20 μL Coomassie brilliant blue G-250 dye, 80 μL water, and 100 μL supernatant were incubated for 10 min in a 96-well plate. The standard curve was created using 20 μL Coomassie brilliant blue G-250 dye, *x* μL (*x* = 20, 40, 60, 80, and 100) BSA solution (0.5 mg mL^−1^), and (180 − *x*) μL water under the same conditions. The absorbance at 595 nm was measured using a microplate reader (Multiskan FC, Thermo Fisher Scientific, Waltham, USA).

The pH value of the supernatant was adjusted to 4.0 by HCl (1 M). This pH value was determined based on the protein content after acid precipitation (Fig. S1†). After 1 h of incubation, the RAS protein was collected by centrifugation and vacuum dried. The yield of RAS protein was calculated as follows:1
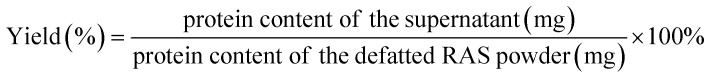


A series of single-factor experiments were conducted to investigate the impact of different factors on the yield of RAS protein. The parameters examined included alkali extraction pH (7.0–12.0), liquid/solid ratio (*e.g.*, the ratio between the extraction solution and the RAS powder, 10 : 1–50 : 1 mL g^−1^), time (0–60 min), and temperature (30–60 °C).

### 2.3 Optimization of the hydrolysis process of RAS protein

The RAS protein powder was suspended in distilled water (liquid/solid ratio = 125 : 1) and then stirred until completely dissolved. Based on the enzymatic conditions in Table S1,† the resulting mixture was heated to the specified temperature and maintained for 20 minutes. After hydrolysis, the enzyme was deactivated by immersing the mixture in a water bath at 100 °C for 20 minutes. The resulting hydrolysate was cooled to room temperature (25 °C) and then centrifuged at 5000*g* for 30 minutes. The supernatant was filtered through a 0.45 μm membrane and stored at −20 °C for later use. The degree of hydrolysis (DH) was used to evaluate the rate of hydrolysis.^23^ It was defined as the number of cleaved peptide bonds over the total number of peptide bonds presented in the sample, and was calculated using the following equation:2
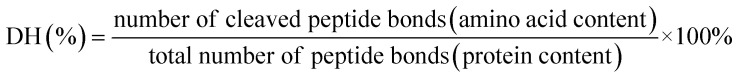
Here, the amino acid content was measured *via ortho*-phthaldialdehyde assay using serine as a standard.

The hydrolysis conditions were investigated through single-factor experiments, including hydrolysis time (0–8 h), temperature (30–70 °C), enzyme/substrate ratio (500–4500 U g^−1^), and liquid/solid ratio (25 : 1–250 : 1 mL g^−1^).

### 2.4 Molecular weight (MW) distribution of RAS protein hydrolysates (RPHs) and RASPs

Crude RPHs (10 mg mL^−1^) were subjected to centrifugal ultrafiltration with MW cut-offs of 10 kDa and 3 kDa, yielding three fractions: RPH-1 (MW > 10 kDa), RPH-2 (3 kDa < MW < 10 kDa), and RPH-3 (MW < 3 kDa). After assessing their antioxidant activity (Fig. S2†), RPH-2 and RPH-3 were combined to obtain final RPHs. They were purified by passing through a Sephadex G-25 gel filtration chromatography column (S7125, Macklin Inc., Shanghai, China), which was eluted with deionized water at a flow rate of 0.5 mL min^−1^ and monitored at 220 nm using a UV/Vis detector (Unico 2800, Unico, Shanghai, China). To determine the MW distribution of RASPs, we used a standard curve (including BSA, insulin, vitamin B-12, and GSH) to correlate the elution volume with log MW.

### 2.5 Identification of RASPs

The purified fraction was freeze-dried to obtain RASPs for solid-phase extraction (SPE). RASPs were dissolved in deionized water (2 mg mL^−1^) and desalted by an SPE C18 column (WAT043395, Waters Corporation, MA, USA). The mixture was eluted with 80% acetonitrile (v/v) and then lyophilized for UHPLC-ESI-Q-TOF-MS/MS analysis.

The acetonitrile solution of RASPs (2 mg mL^−1^) was passed through a 0.22 μm filter. The Shimadzu UHPLC LC-30AD system (Kyoto, China) was achieved on a Luna Omega C18 column (2.1 mm i.d. × 100 mm, 1.6 μm, Phenomenex Inc., Torrance, CA, USA) with a gradient elution of mobile phase A (acetonitrile) and phase B (0.1% formic acid water) at a flow rate of 0.2 mL min^−1^, and the column temperature was maintained at 45 °C. The injection volume was 10 μL, and the gradient elution program was as follows: 0–15 min, 93 → 70%; 15–25 min, 70 → 20% B; 25–35 min, 20% B; 35–36 min, 20 → 93% B; 36–45 min, 20 → 93% B.

The chromatogram was combined with an AB Sciex 5600^+^ Triple-TOF mass spectrometer (AB Sciex Pte. Ltd, Framingham, USA). MS detection was performed in positive electrospray ionization mode, and the MS/MS spectra were obtained in the range of *m*/*z* 50–2000. The final optimized mass parameters were set as follows: ion spray voltage, −4500 V; ion source heater temperature, 450 °C; curtain gas pressure, 30 psi; nebulizing gas pressure, 50 psi; Tis gas pressure, 50 psi; delustering potential, −100 V; collision energy, −45 V; collision energy spread, 15 V. Based on the MS data, RASPs were identified based on *de novo* sequencing using PEAKS Studio 11 program. The search parameters were as follows: 10 ppm MS tolerance; 0.02 Da MS/MS tolerance; fixed modification, carbamidomethylation of Cys; variable modification, oxidation of Met; *de novo* score, ≥50.

### 2.6 Antioxidant evaluation assays

Antioxidant evaluation assays included DPPH˙-scavenging and ABTS˙^+^-scavenging. The DPPH˙-scavenging activity was determined as previously described.^24^ Briefly, 80 μL of DPPH˙-methanolic solution (0.1 M) was mixed with the sample (*x* μL, *x* = 2, 4, 6, 8, and 10) and (20 − *x*) μL of methanol. The mixture was maintained at room temperature (25 °C) for 30 min, and the absorbance was measured at 519 nm on the microplate reader. The percentage of DPPH˙-scavenging activity was calculated based on eqn (3):3
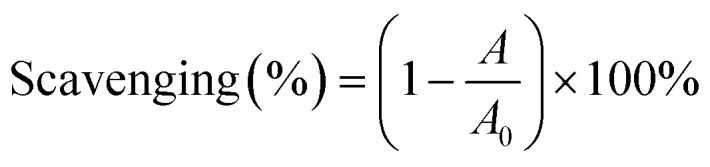
where *A*_0_ is the absorbance at 519 nm of the control and *A* is the absorbance at 519 nm of the test.

The ABTS^+^˙-scavenging activity was evaluated according to a previous method.^24^ Briefly, ABTS^+^˙ was produced by mixing 0.2 mL of ABTS diammonium salt (7.4 mM) with 0.2 mL of potassium persulfate (2.6 mM). The mixture was incubated in the dark at room temperature (25 °C) for 12 h to generate ABTS^+^˙ and then diluted with distilled water. To determine the scavenging activity, *x* μL of sample solution (*x* = 2, 4, 6, 8, and 10) was added to (20 − *x*) μL of distilled water and treated with 80 μL of ABTS^+^˙ reagent. The absorbance at 734 nm was measured after 3 min using distilled water as the blank. The percentage of ABTS^+^˙-scavenging activity was calculated using eqn (3), wherein *A*_0_ is the absorbance at 734 nm of the control (without test agent) and *A* is the absorbance at 734 nm of the reaction mixture (with the sample).

### 2.7 Quantum chemical calculations

Quantum chemical calculations were performed based on the aqueous solvation model based on density using Gaussian 16 program.^25,26^ The geometry optimization and vibration frequency of all stationary points were calculated using the B3LYP-D3(BJ) hybrid function in conjunction with the 6-311G(d,p) basis set.^27–29^ The optimized structures at the local minimum were ensured by the absence of an imaginary frequency. Single-point energy calculations of the optimized geometries were further performed at the M06-2X-D3/def2-TZVP level.^28,30,31^ All output files were visualized and analysed using GaussView 6, VMD 1.9.3, and Multiwfn 3.8 programs.^32–34^ The calculation formulas for the bond dissociation enthalpy (BDE) and ionization potential (IP) were shown in eqn (4) and (5):

4 BDE = *H*(ArO˙) + *H*(H˙) − *H*(ArOH)

5 IP = *H*(ArOH^+^˙) + *H*(e^−^) − *H*(ArOH)

where *H* is the enthalpy value at 298 K. The calculated *H*(e^−^) value in water was obtained from the literature.^35^ The remaining *H* values were calculated as the sum of the thermal correction to enthalpy and the single-point energy.

### 2.8 Molecular docking and molecular dynamics (MD) simulation

The crystal structure of human Keap1 complexed with a 16-mer peptide of Nrf2 was downloaded from the RCSB Protein Data Bank (PDB ID: 2FLU). AutoDock Tools 1.5.6 program was used for protein optimization, removal of water, addition of polar hydrogen and geisteiger charges. AutoDock Vina 1.2.0 program was used for docking jobs.^36^ The coordinates of the original ligand were taken as a grid center (*X* = 6.749, *Y* = 10.143, *Z* = 3.009), and the chosen grid had a size of 22 × 24 × 30 points.

The 3D conformation of ligand–protein complex was extracted from the docking results for MD simulation. The simulation was carried out using Desmond program under the conditions of 300 K and 101.3 kPa. The systems were placed in an octahedral box and solvated using simple point charge water molecules. Sodium ions were added to the box to ensure electroneutrality. The calculations were conducted using an isothermal–isochoric ensemble (NVT) and an isothermal–isobaric ensemble (NPT). The OPLS_2005 force field was used to calculate both the proteins and ligands.^37^ After completing 100 ps of equilibrium calculations, MD simulations were performed for 30 ns. Finally, the trajectories of last 5 ns were analysed for binding free energy (Δ*G*_binding_) based on the MM-GBSA method.^38^ The MD simulation results were visualized and analysed using PyMOL 2.5.4 and VMD 1.9.3 programs.

### 2.9 Anti-ferroptotic evaluation assays

The cytotoxicity of RASPs was evaluated by a CCK-8 assay.^39^ In brief, HT22 cells were seeded at 1 × 10^4^ cells per mL and cultured at 37 °C in a humidified atmosphere containing 5% CO_2_. After adherence, 5 μL of sample solution was added to the culture. After 24 h of incubation, the medium was removed, and the cultures were washed twice with PBS. Then, 100 μL of CCK-8 solution was added to each well and incubated for 2 h. Finally, the absorbance was measured at 450 nm, and the solution containing 10% CCK-8 (without cells and samples) was used as a blank to calculate the percentage of cell viability.

The erastin-induced ferroptosis model was used to evaluate the anti-ferroptotic effect of RASPs on HT22 cells. Cell viability and LPO levels were measured using CCK-8 assay and C11-BODIPY assay, respectively.^40^ In the CCK-8 assay, HT22 cells were divided into three groups: control group, model group, and sample group. The control group received only medium, the model group received erastin, and the sample group received erastin with RASP or Fer-1. In the C11-BODIPY assay, HT22 cells were seeded into 12-well plates at a density of 1 × 10^5^ cells per well and incubated for 24 h to adherence. The control group was incubated in Stel Basal medium for 12 h, while the model and sample groups were exposed to erastin. After medium removal, the cells were then analysed using a C11-BODIPY sensor (2.5 μM), and images were acquired using a fluorescence microscope (BX53, Olympus Corporation, Tokyo, Japan).

### 2.10 Western blot and immunofluorescence analysis of Nrf2 in HT22 cells

In western blot analysis, HT22 cells were seeded into 6-well plates at a density of 5 × 10^5^ cells per well and incubated for 24 h to adherence. The control group was incubated with Stel Basal medium for 12 h, while the model and sample groups were exposed to erastin. Total cellular lysates and nuclear fractions from the treated HT22 cells were obtained using ice-cold RIPA lysis buffer with protease inhibitors and NE-PER nuclear extraction reagent, respectively. The protein concentration in the supernatant was determined using the BCA kit. Equivalent amounts of cell lysates (40 μg) were resolved by 12% SDS-PAGE and electrophoretically transferred to a PVDF membranes. Membranes were blocked with 5% nonfat milk in Tris-buffered saline containing 0.1% Tween 20 for 0.5 h to block nonspecific sites, followed by incubation with primary antibodies against Nrf2 (AF0639, Affinity, Changzhou, China) and Lamin B1 (AF5161, Affinity, Changzhou, China) for 12 h at 4 °C. After washing with Tween 20 three times, fluorescein-conjugated goat anti-rabbit IgG (H + L) secondary antibody (ab216777, Abcam, Shanghai, China) was added and incubated for 1 h at 37 °C. An enhanced chemiluminescence detection was used to visualize the immunoreactive protein signals.^41^

In immunofluorescence analysis, HT22 cells were seeded into 24-well plates at a density of 5 × 10^4^ cells per well and incubated for 24 h to adherence. The control group was incubated with Stel Basal medium for 12 h, while the model and sample groups were exposed to erastin. The treated HT22 cells in various groups were carefully washed three times with PBS. The cells were fixed with 4% paraformaldehyde, and incubated with primary antibodies (AF0639, Affinity, Changzhou, China) overnight at 4 °C. Following washing, the cells were incubated with secondary antibodies and fluorescein-conjugated goat anti-rabbit IgG (H + L) (ab216777, Abcam, Shanghai, China) for 1 h. After incubation, the slides were washed with PBS and treated with DAPI for 10 min. Then, they were rinsed and covered with fluorescent mounting medium. The fluorescence intensity was measured with a confocal laser scanning microscope (LMS800, Zeiss, Oberkochen, Germany).^41^

### 2.11 Statistical analysis

Statistical analysis was conducted using SPSS 26.0 software. Data normality was assessed using the Shapiro–Wilk test. Normally distributed continuous variables were presented as the mean ± standard deviation (SD). For comparisons between two groups, an independent-sample *t* test or *Pearson*'s chi-square test was used. To compare multiple groups, one-way analysis of variance (ANOVA) was used. *Pearson*'s correlation test was utilized to assess the correlation between different measurements. Statistical significance was set at *P* < 0.05. The IC_50_ value was defined as the final concentration of 50% activity percentage and calculated *via* dose–response curves, which were plotted using Origin 2017 program.

## 3. Results and discussion

### 3.1 Optimization of the extraction process of RAS protein

The results in Fig. S3† indicate that pH, liquid/solid ratio (mL g^−1^), and extraction time (min) have significant impacts on the yield (%) of RAS protein. Therefore, we employed an RSM coupled with BBD to optimize the extraction process of RAS protein. The levels of each variable and corresponding yield are presented in Tables S2 and S3.† Accordingly, we utilized Design-Expert 12 program to derive a mathematical equation describing the yield of RAS protein, which is shown as eqn (6):

6 *Y* = 31.76 + 3.03*A* + 0.9768*B* + 0.7671*C* + 1.75*AB* − 0.9590*AC* − 0.4188*BC* − 1.6*A*^2^ + 0.4133*B*^2^ + 0.3001*C*^2^

Here, *Y* is the yield (%) of RAS protein, while *A*, *B*, and *C* are pH, liquid/solid ratio (mL g^−1^), and time (min), respectively.

**Fig. 1:**
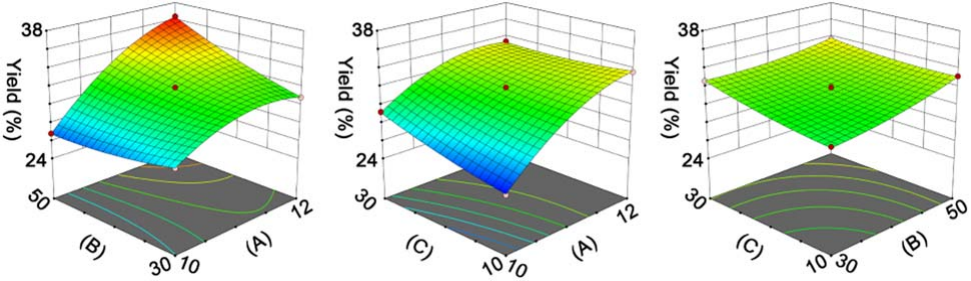
Response surface plot indicating the effect of pH (A), liquid/solid ratio (B, mL g^−1^), and extraction time (C, min) on the yield (%) of RAS protein.

The ANOVA summary for the model shows that the quadratic model is the fittest for the yield of RAS protein, according to the *P* value (*P* < 0.05), lack of fit (*P* > 0.05), and *R*^2^ value (0.9984) (Table S4†). The response surface analysis indicates that the optimal conditions for the extraction of RAS protein are as follows: pH, 11.74; liquid/solid ratio, 49.82 : 1 mL g^−1^; extraction time, 20.17 min (Fig. 1). Under the optimal conditions, the model predicted that the maximum yield of RAS protein was 36.42%, while the actual yield was 36.03% ± 0.31%. The high consistency suggests that the RSM model performs reliably and accurately.

### 3.2 Optimization of the hydrolysis process of RAS protein

To select an appropriate enzyme for hydrolysing RAS protein, DH and free radical scavenging activity were evaluated. Fig. S4 and S5† indicate that the hydrolysates obtained *via* neutral protease simultaneously show higher DH and free radical scavenging activity. Once the optimal enzyme was identified, the factors affecting the DPPH˙-scavenging percentage (%) of hydrolysates, such as hydrolysis time (h), temperature (°C), enzyme/substrate ratio (U g^−1^), and liquid/solid ratio (mL g^−1^) were considered (Fig. S6, Tables S5 and S6†). Using RMS-BBD analysis, a mathematical equation was given as eqn (7):

7 *Y* = 44.84 − 1.34*A* + 2.71*B* + 4.35*C* + 4.70*D* − 3.53*AB* + 5.3*AC* + 6.36*AD* + 0.7377*BC* + 2.47*BD* + 1.57*CD* − 2.17*A*^2^ − 3.2*B*^2^ − 5.73*C*^2^ − 6.92*D*^2^

Here, *Y*, *A*, *B*, *C*, and *D* are the DPPH˙-scavenging percentage (%), reaction time (h), temperature (°C), enzyme/substrate ratio (U g^−1^), and liquid/solid ratio (mL g^−1^), respectively.

**Fig. 2:**
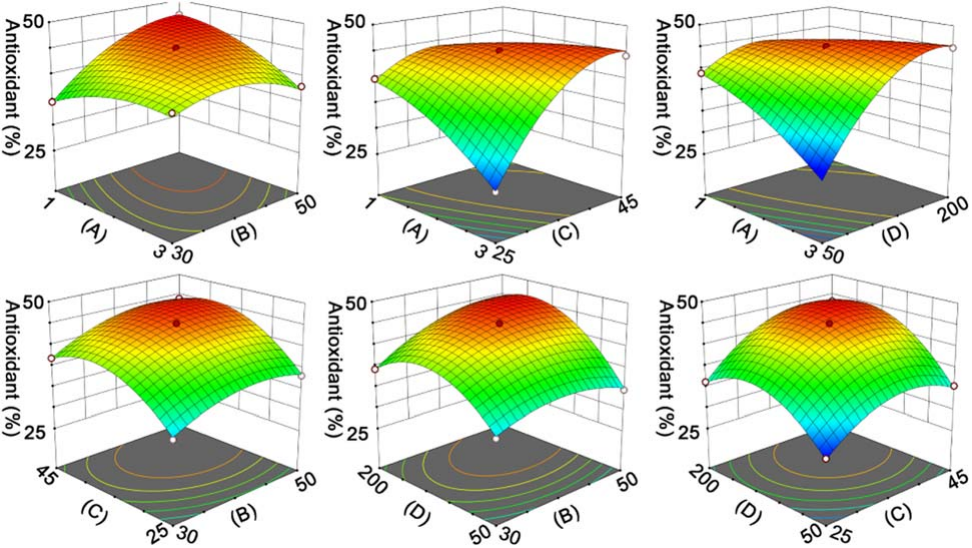
Response surface plot indicating the effect of hydrolysis time (A, h), temperature (B, °C), enzyme/substrate ratio (C, U g^−1^), and liquid/solid ratio (D, mL g^−1^) on the antioxidant activity (%) of RPHs.

The ANOVA results in Table S7† indicate the rationality of the quadratic model. According to the RSM analysis (Fig. 2), the optimal conditions for the hydrolysis process are as follows: hydrolysis time, 2.58 h; temperature, 43.37 °C; enzyme/substrate ratio, 4195.16 U g^−1^; liquid/solid ratio, 169.78 : 1 mL g^−1^. The model predicted a DPPH˙-scavenging percentage of 49.46%, and the actual activity was found to be 48.43% ± 0.52%.

### 3.3 Molecular weight distribution analysis of RPHs

**Fig. 3:**
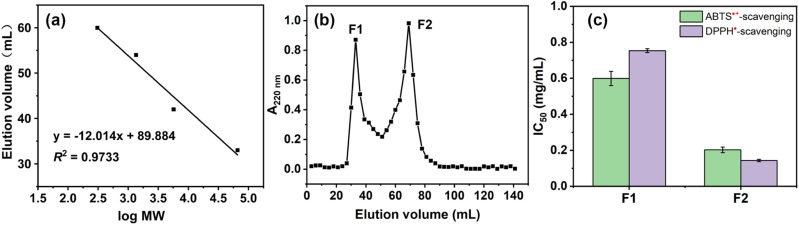
(a) Curve of standards (including bovine serum albumin, insulin, vitamin B-12, and glutathione) between elution volume (mL) and log MW. (b) Chromatogram for RPHs of gel filtration chromatography. (c) DPPH˙- and ABTS˙^+^-scavenging activities of F1 and F2.

The hydrolysates were concentrated and lyophilized to obtain RPHs for subsequent analysis. The RPHs exhibit apparent antioxidant ability in both aqueous and organic solvents, indicating the potential antioxidant and anti-ferroptotic effects of RASPs (Fig. S7†). It is widely recognized that MW distribution is a crucial factor that influences the functional properties of protein hydrolysates.^42^ To demonstrate this, two fractions were collected from RPHs and labelled F1 and F2. The MWs of F1 and F2 were determined by SPE analysis using the calibration curve equation of log MW and elution volume (Fig. 3a), Based on the peak area and calibration curve equation (Fig. 3a and b), the MWs of F1 and F2 were observed to be above 1.72 kDa and below 0.97 kDa, respectively. In different antioxidant assays (Fig. 3c), F2 shows stronger activity than F1, suggesting that MW affects the antioxidant action of RPHs, and F2 is appropriate for the isolation and identification of RASPs.

### 3.4 Identification and screening of antioxidant RASPs

UHPLC-ESI-Q-TOF-MS/MS analysis was performed to identify RASPs from F2. The results are depicted in Fig. S8.† RASPs with the top 50 peak areas were identified and then subjected to docking analysis with Keap1 (Table S8†).

**Fig. 4:**
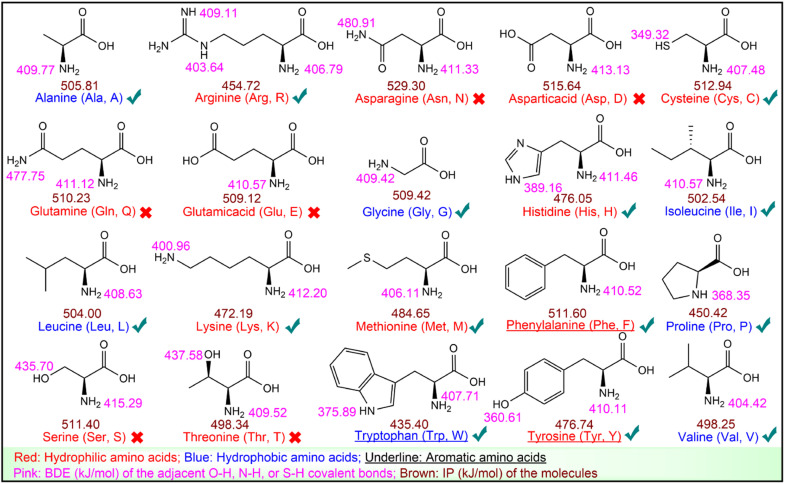
BDEs and IPs of the 20 common amino acids. The “√” and “×” represent the antioxidant amino acids and non-antioxidant amino acids, respectively.

From a chemical perspective, the peptide–Keap1 complex is formed through weak interactions, such as hydrogen bonds and hydrophobic interactions. Therefore, hydrophobic and aromatic amino acids are considered as antioxidant residues.^43^ The antioxidant activity of the 20 common amino acids was further tested, showing that Cys, Tyr, Trp, Phe, Met, His, Lys, and Arg effectively scavenged DPPH˙ or ABTS˙^+^ (Table S9†). The two free radicals are known to be eliminated through hydrogen atom transfer (HAT) and electron transfer (ET) mechanisms, which are characterized by BDE and IP, respectively.^24^ They were calculated using quantum chemistry, as shown in Fig. 4.

Fig. 4 suggests that antioxidant amino acids have lower BDEs or IPs. Particularly, among the eight antioxidant amino acids, Cys, Met, His, Lys, and Arg are neither hydrophobic nor aromatic amino acids, but they display a low BDE or IP, which enables them to scavenge DPPH˙ or ABTS˙^+^. These results imply that in addition to hydrophobic and aromatic amino acids, amino acids with low BDE or low IP should also be considered as antioxidant residues (Fig. 4). Thus, all 50 RASPs contain at least one antioxidant residue, indicating their promising Keap1–Nrf2 PPI inhibitory effect.

In this research, potential Keap1–Nrf2 PPI inhibitory RASPs were selected based on the antioxidant residue, peak area, and binding affinity. Accordingly, the top 10 RASPs in Table S8† were synthesized for further investigation. Their MS data are presented in Fig. S9–S18.†

### 3.5 Antioxidant effect of RASPs

DPPH˙-scavenging and ABTS˙^+^-scavenging assays prove the HAT- and ET-based antioxidant effect of RASPs (Table 1). To explore the potential structure-activity relationships, some related theoretical parameters were calculated. Specifically, the highest occupied molecular orbital (HOMO) and the lowest unoccupied molecular orbital (LUMO) energies describe the electron-donating and electron-accepting potential of a molecule, respectively. Their energy gap (*e.g.*, HOMO–LUMO gap) represents the molecular reactivity and stability.^44^ The *E*_HOMO_ and HOMO–LUMO gap (Δ*E*) of 10 RASPs are shown in Table 1. *Pearson* correlation analysis reveals a positive correlation between Δ*E* and IC_50_ and a negative correlation between *E*_HOMO_ and IC_50_ (Table S10†), emphasizing the importance of ET in the antioxidant process.

**Table 1:** Antioxidant activity, *E*_HOMO_ and Δ*E* of the 20 common amino acids^a^

No.	Sequence	DPPH˙-scavenging (mM)	ABTS˙^+^-scavenging (mM)	*E*_HOMO_ (eV)	Δ*E* (eV)
1	MFQGF	1.88 ± 0.12	4.32 ± 0.28	−6.0385	5.3624
2	FQGF	3.21 ± 0.11	4.75 ± 0.43	−6.7324	6.1580
3	VLPQL	3.85 ± 0.04	7.14 ± 0.12	−6.7301	6.2052
4	FLLP	4.07 ± 0.17	7.20 ± 0.37	−6.5904	5.9342
5	FVTP	3.55 ± 0.27	6.09 ± 0.35	−6.5519	5.9282
6	LLGY	1.41 ± 0.02	1.48 ± 0.01	−6.1371	5.4732
7	LYN	3.14 ± 0.25	3.15 ± 0.33	−6.1833	5.4667
8	LAY	3.15 ± 0.18	5.07 ± 0.36	−6.2443	5.6244
9	TVTY	2.91 ± 0.04	4.09 ± 0.27	−6.2335	5.4248
10	VTGGSYG	2.31 ± 0.11	5.05 ± 0.32	−6.1722	5.3887

a Δ*E* (*E*_LUMO_ − *E*_HOMO_), *E*_LUMO_, and *E*_HOMO_ of the optimized molecules were calculated under the level of M06-2X-D3/def2-TZVP. The radical scavenging activity was evaluated using IC_50_.

It is noteworthy that the HOMOs of 10 RASPs are primarily located around the antioxidant residues mentioned earlier, including Met, Phe, Val, and Tyr (Fig. S19†). In addition, the HOMO of a peptide and the HOMO of the corresponding antioxidant amino acid are quite similar. These findings underscore the critical role of antioxidant residues in RASPs and imply that the antioxidant sites of amino acids remain unchanged after peptide formation. However, the present results cannot explain the relative activity between the 10 RASPs, which requires further investigation.

### 3.6 Anti-ferroptotic effect of RASPs

To evaluate the cytotoxicity of the 10 RASPs, a CCK-8 assay was performed. The results demonstrate that neither F2 nor RASPs exhibit any significant cytotoxic effect at high concentrations (500 μg mL^−1^ and 1000 μM) after 24 h of incubation with HT22 cells (Fig. S20†), which are known to possess cholinergic properties and serve as an *in vitro* model for investigating neurodegenerative diseases.^45^ The low cytotoxicity highlights the potential application of RASPs in treating ferroptosis-related diseases.

Fig. S21† shows that erastin, a classic inducer of ferroptosis, reduces the viability of HT22 cells in dose- and time-dependent manners, with an approximate half-maximal inhibition (52.60% ± 4.78%) observed at 10 μM after 24 hours of incubation.^1^ Under this incubation condition, erastin reduced cell counts, inhibited neurite outgrowth, and induced LPO accumulation in HT22 cells (Fig. S22a and b†). However, cotreatment with erastin and Fer-1 improved cell morphology and reduced LPO level (Fig. S22c†). These results indicate that the ferroptotic model of HT22 cells is successfully established and can be inhibited *via* the Keap1–Nrf2 PPI inhibitor.

**Fig. 5:**
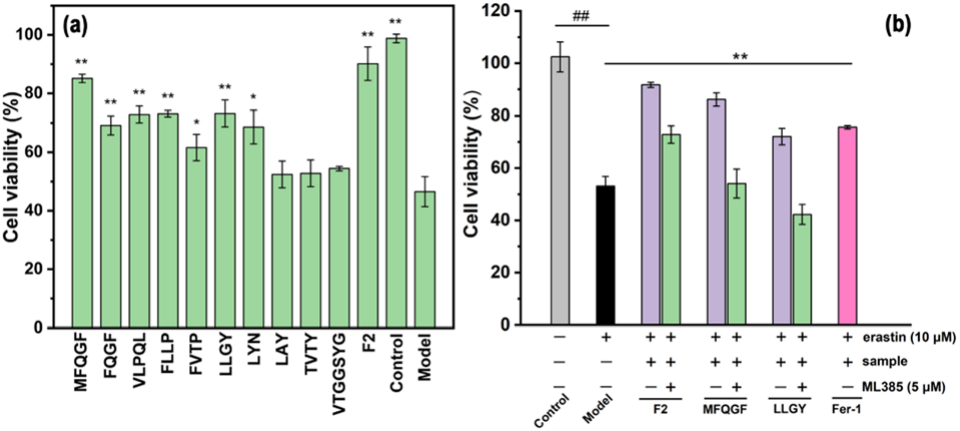
(a) The anti-ferroptotic effect of F2 and RASPs on HT22 cells. *P < 0.05, **P < 0.01, significant difference from the model group. (b) The effect of ML385 on the anti-ferroptotic ability of F2 (40 μg mL^−1^) and RASPs (125 μM). ## or **, significant difference from the model group (*P* < 0.01).

**Fig. 6:**
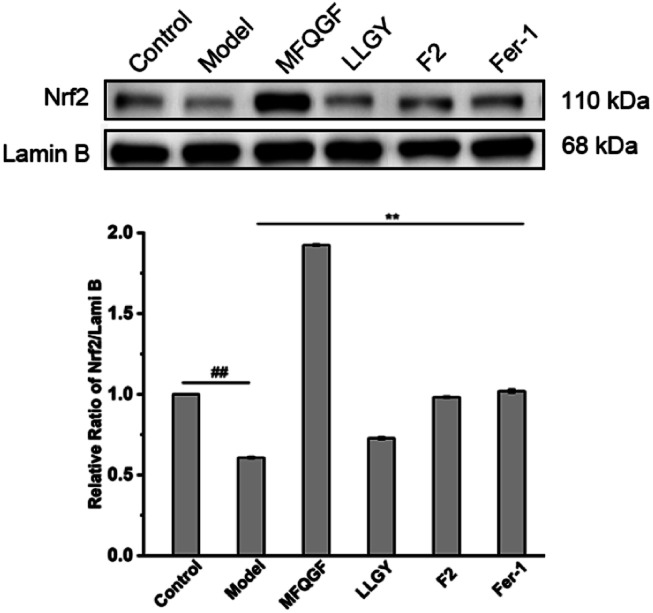
Western blot analysis of Nrf2 in HT22 cells (## or **, significant difference with model group *P* < 0.01). The raw data of western blots were shown in Fig. S23.†

**Fig. 7:**
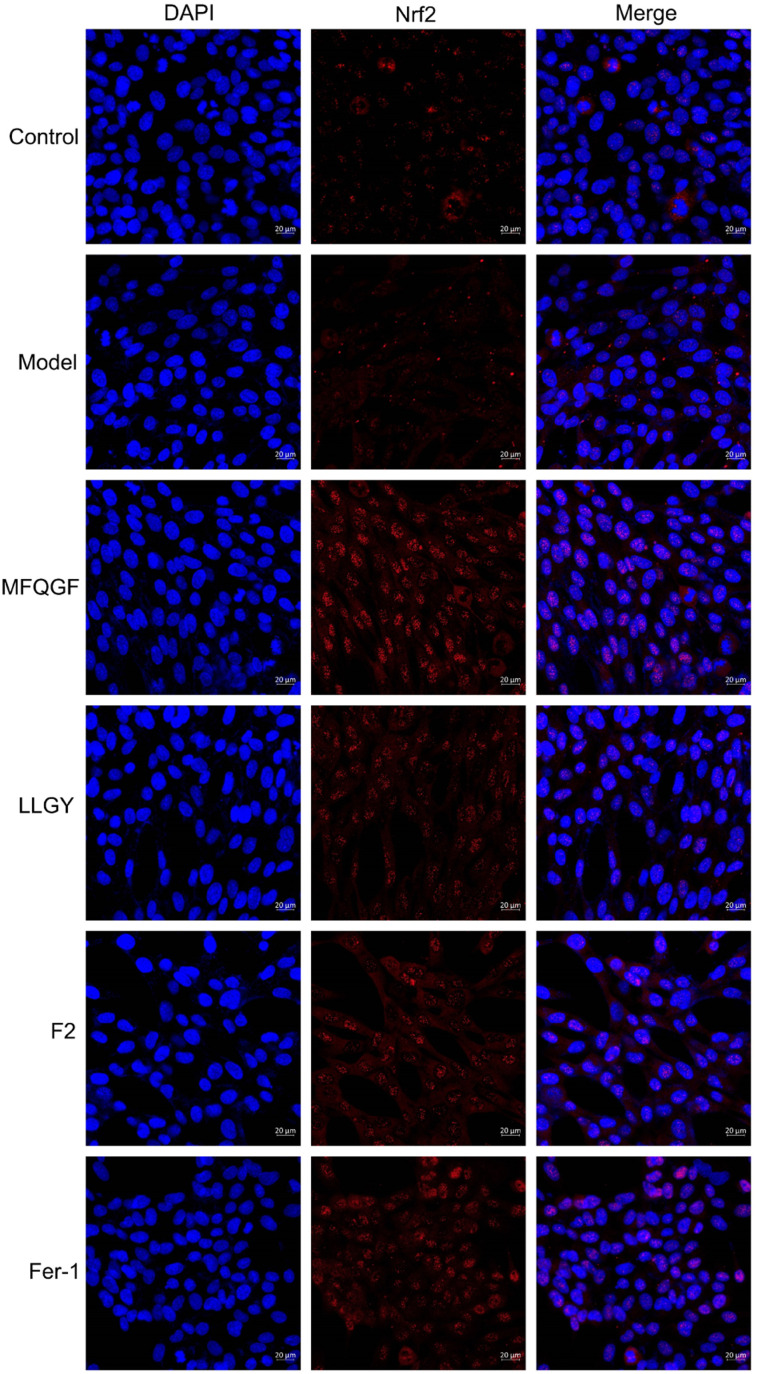
Immunofluorescence staining of HT22 cells. The model and sample groups were treated with erastin.

The CCK-8 assay confirms the anti-ferroptotic effect of F2 and 10 RASPs (Fig. 5a). To explore the relationship between anti-ferroptosis and Keap1–Nrf2 PPI inhibition, we selected MFQGF and LLGY, two RASPs that possess higher anti-ferroptotic levels than other RASPs, to incubate with a known Nrf2 inhibitor named ML385.^46^Fig. 5b shows that F2, MFQGF, and LLGY possess great anti-ferroptotic capacity, which can be significantly weakened by ML385. Western blot and immunofluorescence assays show that RASPs upregulate and accumulate Nrf2 in the nucleus of HT22 cells (Fig. 6 and 7). These results suggest that the anti-ferroptotic effect is closely associated with Nrf2 activation.

This study investigated the interactions of MFQGF and LLGY with Keap1 using MD simulation and depicted the stable complexes in Fig. S24.† Accordingly, we calculated the root mean square deviation (RMSD), which enabled us to estimate any structural drifts and alterations in the complexes. The result in Fig. S25† shows that after 10 ns, the complex trajectories display no significant structural differences, indicating that RASPs can form stable bonds with Keap1 within its initial binding pocket. Additionally, we analysed local changes in Keap1 residues using the root mean square fluctuation (RMSF), and the RMSF shows a similarity in the residue fluctuation from the two complexes (Fig. S26†). These results suggest that the intermolecular interactions of the two RASP–Keap1 complexes are similar.

The intermolecular interactions between RASP and Keap1 are presented in Fig. S27 and S28.† Key residues such as Tyr334, Asn382, Asn414, Arg415, Ile461, Ser508, Ala556, Tyr572, and Ser602 of Keap1 are found to play a crucial role in the binding of RASP–Keap1 complexes *via* hydrogen bonds and hydrophobic interactions. MM/GBSA analysis (Table S11†) reveals that MFQGF binds more easily to Keap1 than LLGY, which is consistent with their anti-ferroptotic ability (Fig. 5).

**Fig. 8:**
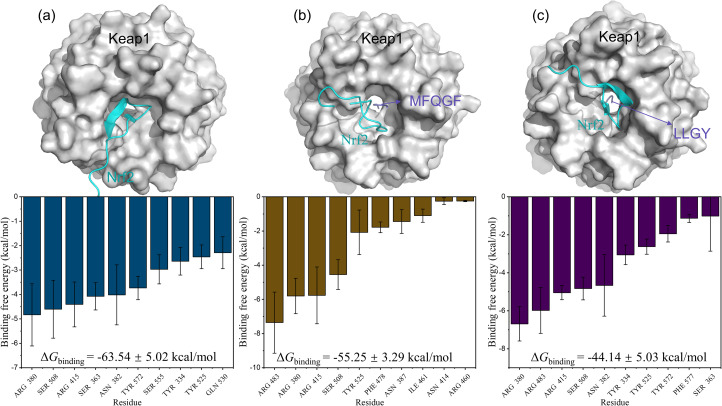
3D conformations with free energy decompositions of Keap1–Nrf2 (a), MFQGF–Keap1–Nrf2 (b), and LLGY–Keap1–Nrf2 (c).

Furthermore, MD simulations of RASP–Keap–Nrf2 ternary complexes were also performed. The results in Fig. 8 show that Nrf2 is mainly bound to Keap1 through residues Arg380, Ser508, Arg415, and Ser363, many of which are also binding sites for RASPs and Keap1. Therefore, RASPs occupy the active pocket of Keap1, dissociate Nrf2, and increase the binding energy of Keap1–Nrf2. Obviously, the MD results suggest that RASPs directly inhibit the Keap1–Nrf2 PPI, indicating the safety and effectiveness of RASPs.

## 4. Conclusions

Through the optimized protocols, at least 50 RASPs could be identified in RAS protein. These RASPs possess antioxidant ability due to the presence of active residues, including Met, Phe, Val, and Tyr. They exert antioxidant ability *in vitro* based on HAT and ET mechanisms. Among them, at least 10 RASPs exhibit anti-ferroptotic effect on HT22 cells, which is attributed to the direct inhibition of Keap1–Nrf2 PPI. Thus, RASPs should be considered as ideal Keap1–Nrf2 PPI inhibitors from medicinal plants for antioxidant and anti-ferroptotic purposes.

## Author contributions

Ban Chen: investigation, software, data curation, writing – original draft, writing – review & editing. Xiaojian Ouyang: conceptualization, writing – original draft, writing – review & editing, supervision, project administration. Chunfeng Cheng: investigation, software, writing – review & editing. Dongfeng Chen: investigation, software. Jiangtao Su: investigation, software. Yuchen Hu: investigation, software. Xican Li: conceptualization, writing – original draft, writing – review & editing, supervision, project administration.

## Conflicts of interest

There are no conflicts to declare.

## Supplementary Material

RA-013-D3RA04057G-s001
