# Supplementary material for: Bioactive peptides derived from Radix Angelicae sinensis inhibit ferroptosis in HT22 cells through direct Keap1–Nrf2 PPI inhibition
Source: RSC Adv. 2023 Jul 24;13(32):22148–57. doi: 10.1039/d3ra04057g (PMC10363710; doi:10.1039/d3ra04057g)
Supplement: RA-013-D3RA04057G-s001 [file RA-013-D3RA04057G-s001.pdf]

Electronic Supplementary Material (ESI) for RSC Advances.
This journal is © The Royal Society of Chemistry 2023

Supplementary Information

Bioactive peptides derived from *Radix Angelicae* Sinensis inhibit ferroptosis in HT22 cells through direct Keap1-Nrf2 PPI inhibition

Ban Chen,[a] Xiaojian Ouyang,*[b] Chunfeng Cheng,[c] Dongfeng Chen,[d] Jiangtao Su,[a] Yuchen Hu[a] and Xican Li*[e]

a. Key Laboratory of Fermentation Engineering (Ministry of Education), Cooperative Innovation Center of Industrial Fermentation (Ministry of Education & Hubei Province), Hubei University of Technology, Wuhan, 430068, China.

b. IncreasePharm Hengqin Inst. Co., Ltd., Zhuhai, 519000, China. E-mail: oyxiaojian55@163.com

c. Shenzhen Hospital of Integrated Traditional Chinese and Western Medicine, Shenzhen, 518000, China.

d. School of Basic Medical Science, Guangzhou University of Chinese Medicine, Guangzhou 510000, China.

e. School of Chinese Herbal Medicine; Guangzhou University of Chinese Medicine, Guangzhou, 510000, China. E-mail: lixican@126.com

Contents

Fig. S1 Protein content of the supernatant after acid precipitation at different pH values. 4

Table S1 Hydrolysis conditions under different proteases..5

Fig. S2 Antioxidant activity of RAS protein hydrolysates (RPHs)..6

Fig. S3 The effects of alkali extraction pH, liquid/solid ratio, time, and temperature on the yield (%) of RAS proteins. 7

Table S2 Three independent variables with their corresponding levels when extracting RAS protein. 8

Table S3 Box–Behnken response surface design scheme and results when extracting RAS protein.........................9

Table S4 ANOVA for response surface quadratic model of extraction process..10

Fig. S4 The degree of hydrolysis (DH) of RAS protein powder under different proteases.....................................11

Fig. S5 The degree of hydrolysis (DH) and radical scavenging activity of RAS protein powder under different proteases. 12

Fig. S6 Different factors on the DPPH•-scavenging activity of RPHs...13

Table S5 Four independent variables with their corresponding levels when hydrolysing RAS protein. 14

Table S6 Box–Behnken response surface design scheme and results when hydrolysing RAS protein....................15

Table S7 ANOVA for the response surface quadratic model of the hydrolysis process. 16

Fig. S7 The antioxidant activity of RPHs. 17

Fig. S8 The total ion chromatogram of F2. 18

Table S8 Information on the top 50 RASPs in peak area..19

Table S9 The antioxidant activity, E_{HOMO} and ΔE of 20 common amino acids. 21

Fig. S9 The MS/MS data of RASP MFQGF..22

Fig. S10 The MS/MS data of RASP FQGF. 23

Fig. S11 The MS/MS data of RASP VLPQL..24

Fig. S12 The MS/MS data of RASP FLLP. 25

Fig. S13 The MS/MS data of RASP FVTP...26

Fig. S14 The MS/MS data of RASP LLGY..27

Fig. S15 The MS/MS data of RASP LYN. 28

Fig. S16 The MS/MS data of RASP LAY. 29

Fig. S17 The MS/MS data of RASP TVTY...30

Fig. S18 The MS/MS data of RASP VTGGSYG. 31

Table S10 *Pearson* correlation analysis between IC_{50}, E_{HOMO}, and ΔE (r, P). 32

Fig. S19 HOMOs of the 10 RASPs (a) and antioxidant amino acids (b)...33

Fig. S20 HT22 cell viability under incubation with F2 fraction (500 μg/mL) or RASPs (1000 μM) for 24 h..........34

Fig. S21 HT22 cell viability under the incubation of erastin at various concentrations and times............................35

Fig. S22 Effect of erastin on HT22 cell morphology (a1~c1, 100X) and intracellular LPO content (a2~c2, 200X). 36

Fig. S23 Raw data of Western blots. 37

Fig. S24 3D conformation of MFQGF-Keap1 and LLGY-Keap1 obtained from MD simulation. 38

Fig. S25 RMSD of MFQGF-Keap1 (a) and LLGY-Keap1 (b) derived from MD simulation. 39

Fig. S26 RMSF of MFQGF-Keap1 (a) and LLGY-Keap1 (b) derived from MD simulation. 40

Fig. S27 Interaction mode of the MFQGF-Keap1. 41

Fig. S28 Interaction mode of the LLGY-Keap1. 42

Table S11 Binding free energies and energy components predicted by MM/GBSA (kcal/mol)..............................43

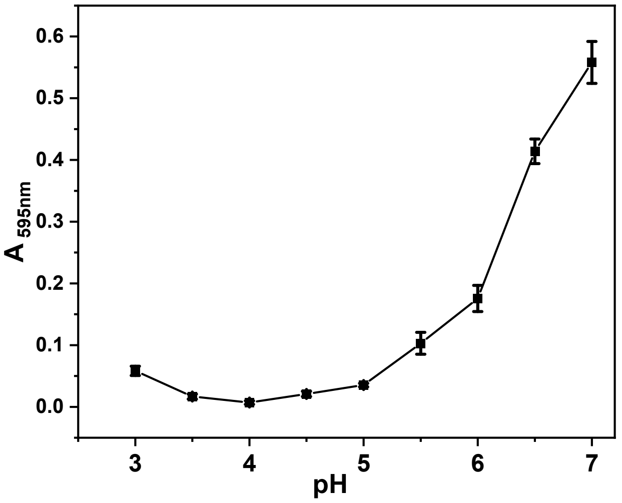

Fig. S1 Protein content of the supernatant after acid precipitation at different pH values.

The content was determined using the Bradford assay, and it was evaluated using the absorbance at 595 nm (A_{595}).

Table S1 Hydrolysis conditions under different proteases.

Protease	Enzyme activity (U/g)	pH	Enzymatic temperature (°C)	Enzymatic time (h)	Amount of enzyme added (U/g)
Alkaline protease	2.0×10^{5}	10.5	45	6	2500
Acid protease	5.0×10^{4}	3.5	50	6	2500
Neutrase	5.0×10^{4}	6.5	40	6	2500
Pepsin	2.5×10^{5}	2.5	37	6	2500
Papain	2.0×10^{5}	6.5	55	6	2500
Parenzyme	2.5×10^{6}	7.5	37	6	2500

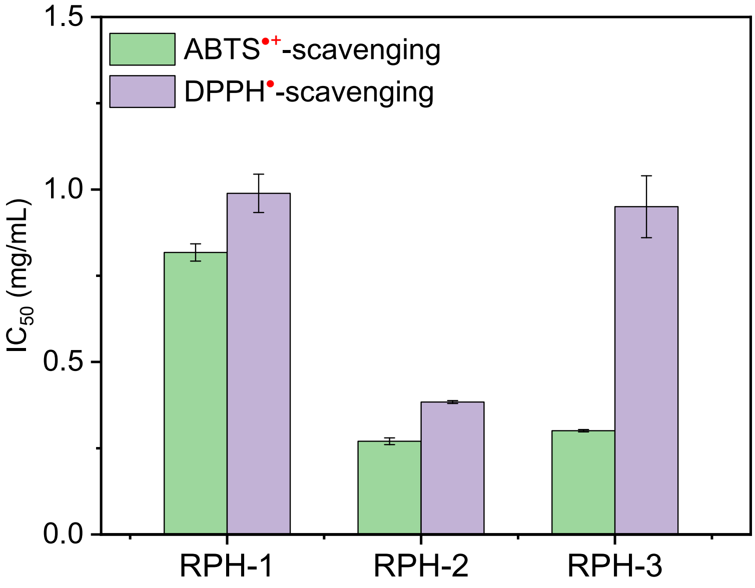

Fig. S2 Antioxidant activity of RAS protein hydrolysates (RPHs).

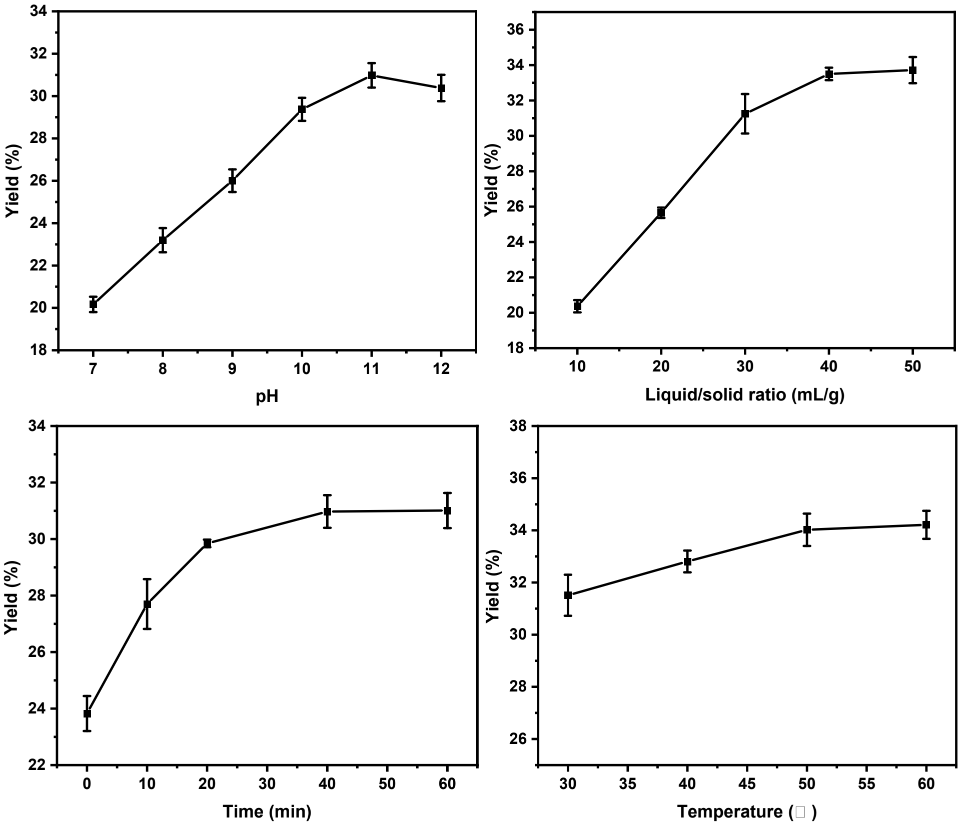

Fig. S3 The effects of alkali extraction pH, liquid/solid ratio, time, and temperature on the yield (%) of RAS proteins.

Table S2 Three independent variables with their corresponding levels when extracting RAS protein.

Level	Independent variables		
	A	B	C
1	10	30:1	10
2	11	40:1	20
3	12	50:1	30

A, alkali extraction pH; B, liquid/solid ratio (mL/g); C, time (min).

Table S3 Box–Behnken response surface design scheme and results when extracting RAS protein.

Run	A	B	C	Yield (%)
1	11	30	30	32.64
2	11	50	30	33.68
3	11	40	20	31.63
4	12	40	30	33.39
5	11	40	20	31.95
6	10	50	20	26.83
7	10	40	30	29.24
8	11	40	20	31.94
9	11	40	20	31.63
10	10	40	10	25.62
11	12	40	10	33.61
12	11	50	10	33.15
13	11	40	20	31.67
14	10	30	20	28.29
15	11	30	10	30.44
16	12	50	20	36.36
17	12	30	20	30.84

A, alkali extraction pH; B, liquid/solid ratio (mL/g); C, time (min).

Table S4 ANOVA for response surface quadratic model of extraction process.

Source	Sum of Squares	*df*	Mean square	*F*	*P*
Model	113.6938	9	12.6327	488.5200	<0.0001
A	73.3684	1	73.3684	2837.2600	<0.0001
B	7.6323	1	7.6323	295.1500	<0.0001
C	4.7078	1	4.7078	182.0600	<0.0001
AB	12.1835	1	12.1836	471.1600	<0.0001
AC	3.6787	1	3.6787	142.2600	<0.0001
BC	0.7014	1	0.7014	27.1200	0.0012
A^2	10.7311	1	10.7312	414.9900	<0.0001
B^2	0.7192	1	0.7192	27.8100	0.0012
C^2	0.3791	1	0.3791	14.6600	0.0065
Residual	0.1810	7	0.0259		
Lack of fit	0.0718	3	0.0239	0.8775	0.5238
Pure error	0.1092	4	0.0273		
Total	113.8748	16	R^2=0.9984	Adj R^2=0.9984	

A, alkali extraction pH; B, liquid/solid ratio (mL/g); C, time (min).

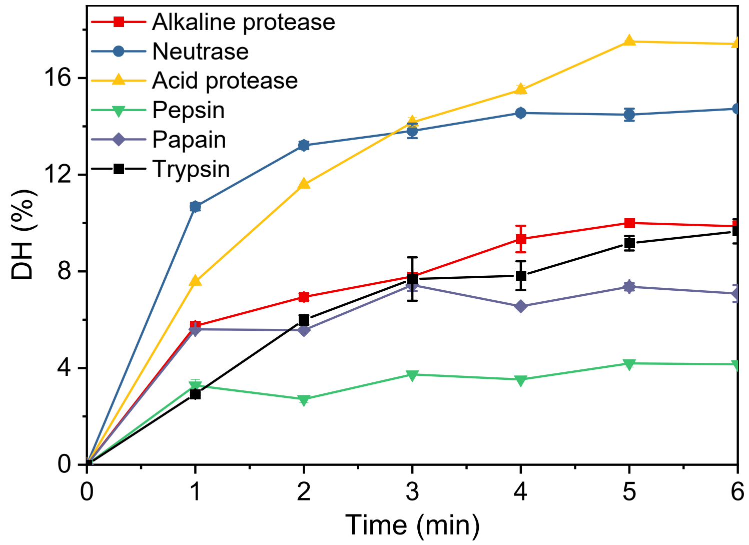

Fig. S4 The degree of hydrolysis (DH) of RAS protein powder under different proteases.

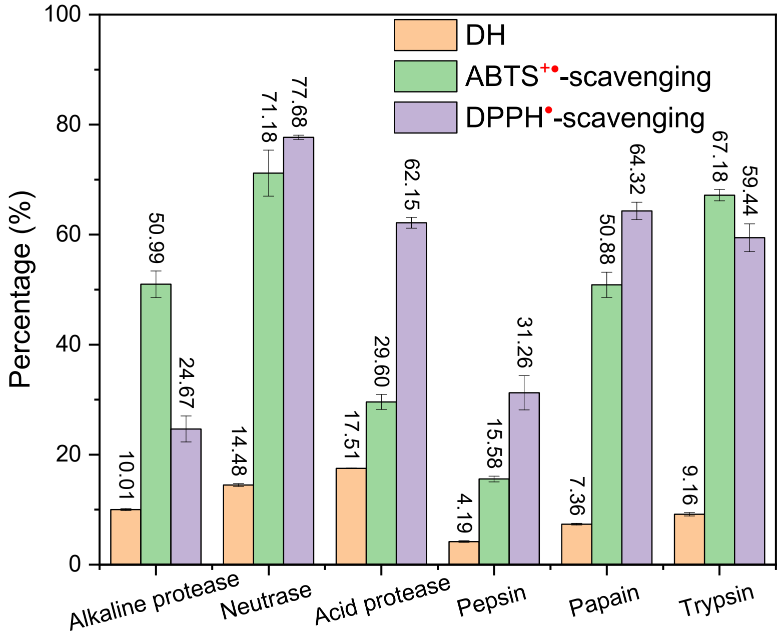

Fig. S5 The degree of hydrolysis (DH) and radical scavenging activity of RAS protein powder under different proteases.

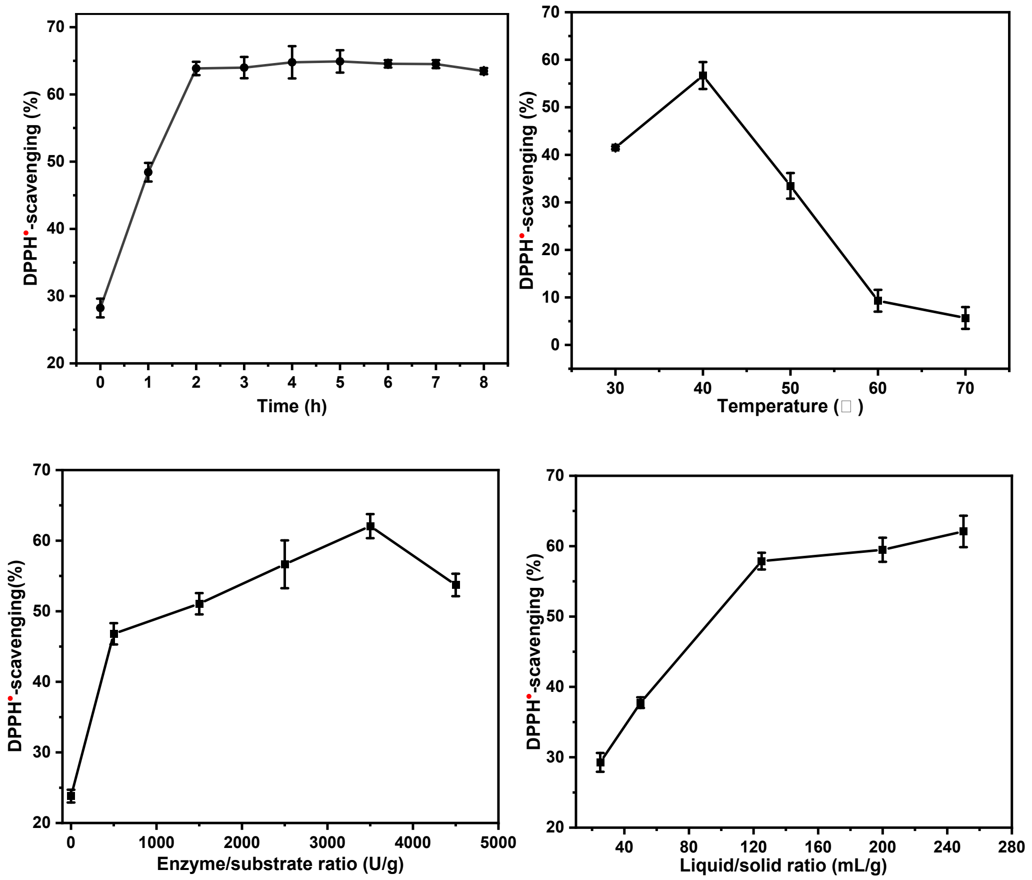

Fig. S6 Different factors on the DPPH•-scavenging activity of RPHs.

The parameters examined included hydrolysis time (0–8 h), temperature (30–70℃), enzyme/substrate ratio (500–4500 U/g), and liquid/solid ratio (25:1–250:1 mL/g).

Table S5 Four independent variables with their corresponding levels when hydrolysing RAS protein.

Independent variables	Level		
	-1	0	1
A	1	2	3
B	30	40	50
C	2500	3500	4500
D	50:1	125:1	200:1

A, time (h); B, temperature (℃); C, enzyme/substrate ratio (U/g); D, liquid/solid ratio (mL/g).

Table S6 Box–Behnken response surface design scheme and results when hydrolysing RAS protein.

Run	A	B	C	D	DPPH$^{\bullet}$ scavenging activity (%)
1	2	40	3500	125	45.19
2	3	40	4500	125	44.34
3	3	40	3500	200	45.02
4	2	50	3500	200	33.95
5	2	30	4500	125	36.88
6	1	40	3500	200	35.14
7	2	40	3500	125	44.34
8	2	30	3500	200	34.28
9	2	40	3500	125	45.15
10	2	50	3500	50	29.78
11	2	30	2500	125	29.11
12	1	40	2500	125	39.67
13	3	50	3500	125	38.01
14	3	40	2500	125	25.79
15	1	40	3500	50	38.91
16	2	40	3500	125	44.34
17	2	40	2500	200	31.22
18	2	50	4500	125	43.89
19	2	30	3500	50	29.33
20	3	40	3500	50	36.54
21	2	40	3500	125	45.19
22	2	40	2500	50	25.00
23	2	40	4500	200	43.27
24	1	40	4500	125	37.02
25	1	50	3500	125	47.00
26	1	30	3500	125	34.62
27	2	40	4500	50	30.77
28	3	30	3500	125	39.74
29	2	50	2500	125	33.17

A, time (h); B, temperature (°C); C, enzyme/substrate ratio (U/g); D, liquid/solid ratio (mL/g).

Table S7 ANOVA for the response surface quadratic model of the hydrolysis process.

Source	Sum of Squares	*df*	Mean square	*F*	*P*
Model	1163.5718	14	83.1123	205.3735	<0.0001
A	17.9644	1	17.9644	44.3907	<0.0001
B	73.1783	1	73.1783	180.8264	<0.0001
C	227.1213	1	227.1213	561.2251	<0.0001
D	183.5889	1	183.5889	453.6551	<0.0001
AB	49.8037	1	49.8037	123.0668	<0.0001
AC	112.3705	1	112.3705	277.6717	<0.0001
AD	100.7929	1	100.7929	249.0631	<0.0001
BC	2.1765	1	2.1765	5.3782	0.0388
BD	15.2139	1	15.2139	37.5942	<0.0001
CD	9.8542	1	9.8542	24.3501	0.0003
A^2	26.7399	1	26.7399	66.0753	<0.0001
B^2	57.9945	1	57.9945	143.3065	<0.0001
C^2	203.3195	1	203.3195	502.4101	<0.0001
D^2	259.5155	1	259.5155	641.2725	<0.0001
Residual	4.8563	12	0.4047		
Lack of fit	4.0190	8	0.5024	2.4001	0.2074
Pure error	0.8373	4	0.2093		
Total	1168.4280	26	R^2=0.9958	Adj R^2=0.9910	

A, time (h); B, temperature (℃); C, enzyme/substrate ratio (U/g); D, liquid/solid ratio (mL/g).

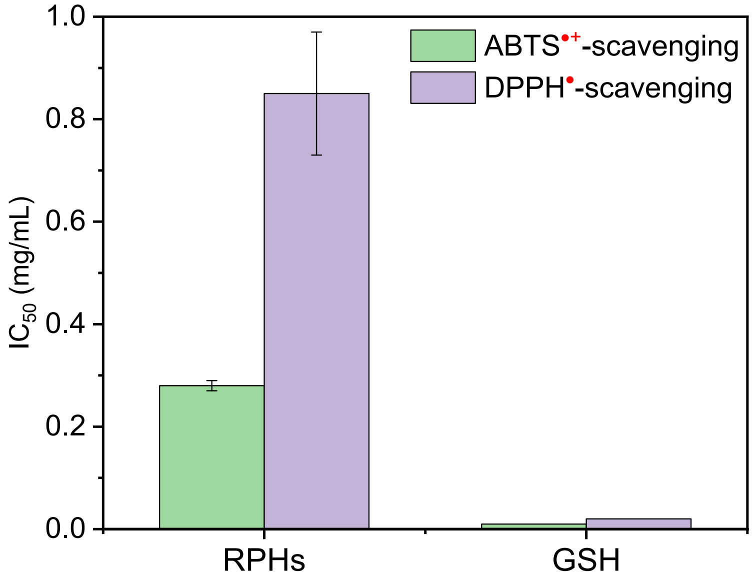

Fig. S7 The antioxidant activity of RPHs.

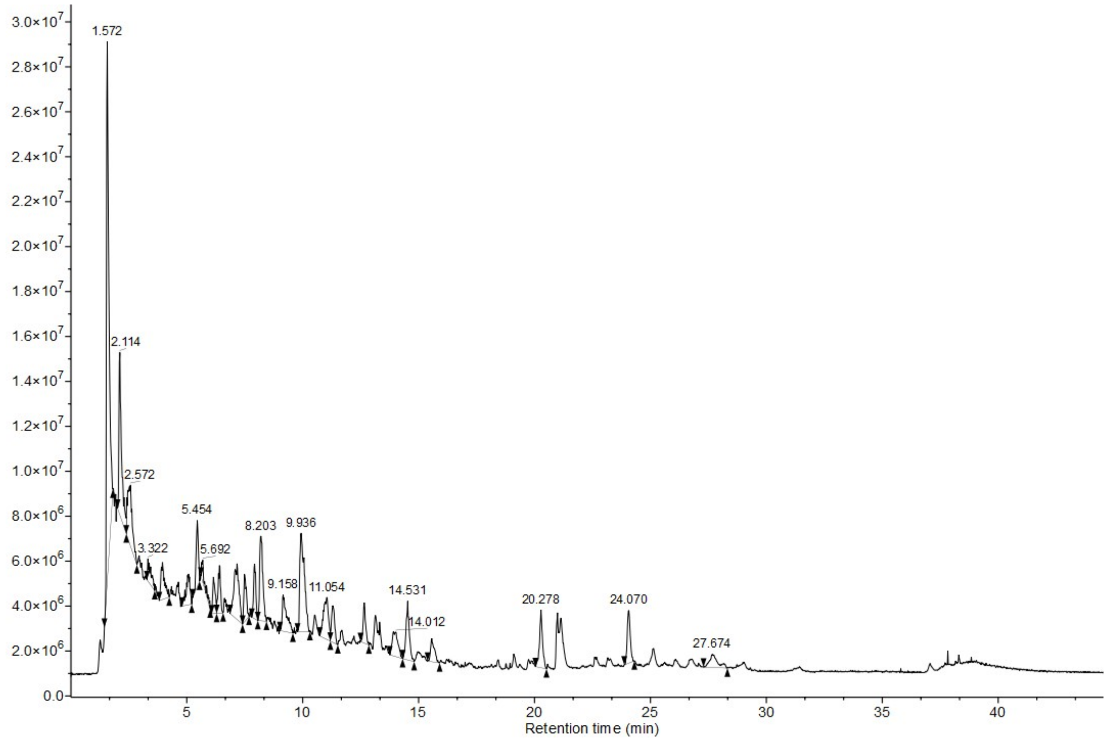

Fig. S8 The total ion chromatogram of F2.

Table S8 Information on the top 50 RASPs in peak area.

No.	Sequence	RT (min)	*m/z*	Peak area	Affinity （kcal/mol）
1	MFQGF	13.32	629.2735	4.58×10^4	-9.28
2	FQGF	10.90	498.2343	9.38×10^4	-9.08
3	VLPQL	12.66	569.3654	1.34×10^5	-8.07
4	FLLP	14.75	489.3063	5.60×10^4	-7.95
5	FVTP	7.11	463.2542	4.33×10^4	-7.9
6	LLGY	10.81	465.2701	3.27×10^4	-7.88
7	LYN	3.36	409.2076	1.15×10^5	-7.78
8	LAY	7.19	366.2022	9.75×10^4	-7.77
9	TVTY	6.17	483.2436	1.31×10^5	-7.65
10	VTGGSYG	3.91	640.2918	5.23×10^4	-7.65
11	VVNQF	7.93	606.3234	1.82×10^5	-7.57
12	LATY	6.39	467.2492	1.08×10^5	-7.47
13	VFL	12.62	378.2383	4.12×10^4	-7.47
14	LAFLP	15.67	560.3437	4.70×10^4	-7.36
15	VLNK	1.57	473.3077	8.00×10^4	-7.32
16	LFSP	9.50	463.2538	3.52×10^4	-7.23
17	LLAN	5.51	430.2649	7.10×10^4	-7.18
18	LW	10.65	318.1808	3.72×10^4	-7.08
19	VYP	5.79	378.2022	3.44×10^4	-7.07
20	FF	11.03	313.1543	8.07×10^4	-7.06
21	VLPGA	7.03	456.2810	3.51×10^4	-7.06
22	LLPQ	7.25	470.2962	4.22×10^4	-7.05
23	FY	7.07	329.1494	3.86×10^4	-7.02
24	LAF	11.33	350.2072	3.58×10^4	-7.01
25	VLPQ	5.43	456.2807	3.20×10^4	-7.03
26	AFK	3.52	365.2184	3.35×10^4	-6.92
27	VLF	12.90	378.2383	3.58×10^4	-6.88
28	LVT	4.61	332.2169	4.54×10^4	-6.88
29	LF	9.16	279.1700	1.11×10^4	-6.85
30	LVL	11.01	344.2543	4.62×10^4	-6.78
31	LLA	5.51	430.2649	7.10×10^4	-6.75
32	VLA	4.64	302.2063	6.00×10^4	-6.72
33	LY	5.71	295.1646	7.48×10^4	-6.72

34	VF	7.09	265.1542	1.04×10^4	-6.70
35	FA	3.99	237.1231	5.74×10^4	-6.67
36	FL	9.94	279.1699	6.76×10^4	-6.63
37	LLM	9.74	376.2254	6.01×10^4	-6.62
38	LLF	14.43	392.2548	4.20×10^4	-6.62
39	TF	2.97	267.1332	5.84×10^4	-6.60
40	VLM	9.30	362.2074	5.94×10^4	-6.57
41	LSTGTHNASQ	1.57	508.2432	3.82×10^4	-6.53
42	LLP	8.62	342.2383	6.62×10^4	-6.52
43	LVA	5.15	302.2070	4.45×10^4	-6.47
44	LVM	8.60	362.2099	5.14×10^4	-6.37
45	LVP	6.51	328.2230	4.51×10^4	-6.20
46	LR	1.57	288.2026	6.24×10^4	-6.10
47	LL	8.20	245.1852	4.68×10^4	-6.08
48	VL	5.63	231.1702	1.43×10^4	-5.83
49	LM	5.79	263.1417	3.48×10^4	-5.78
50	FK	1.57	294.1806	2.63×10^4	-5.70

Table S9 The antioxidant activity, E_{HOMO} and ΔE of 20 common amino acids.

No.	Amino acids	DPPH$^{\bullet}$-scavenging (mM)	ABTS$^{\bullet+}$-scavenging (mM)	E_{HOMO} (eV)	ΔE (eV)
1	Cys (C)	0.06 ± 0.00	0.03 ± 0.00	-6.7572	6.8522
2	Tyr (Y)	1.37 ± 0.30	0.48 ± 0.02	-6.0355	5.6919
3	Trp (W)	9.13 ± 0.64	2.6 ± 0.16	-5.6022	5.0987
4	Phe (F)	16.11 ± 1.54	n.d.	-6.5377	6.1905
5	Met (M)	29.82 ± 1.53	41.9 ± 1.60	-6.0566	6.6569
6	His (H)	32.23 ± 4.04	5.7 ± 0.13	-6.1791	6.8026
7	Lys (K)	n.d.	1.65 ± 0.05	-6.5016	7.2091
8	Arg (R)	n.d.	2.17 ± 0.14	-6.0487	6.7087
9	Asp (D)	n.d.	n.d.	-6.9760	6.9646
10	Leu (L)	n.d.	n.d.	-6.8594	7.6614
11	Glu (E)	n.d.	n.d.	-6.8277	6.8090
12	Val (V)	n.d.	n.d.	-6.7638	7.4337
13	Thr (T)	n.d.	n.d.	-6.8840	7.6705
14	Ala (A)	n.d.	n.d.	-6.8383	7.5386
15	Ser (S)	n.d.	n.d.	-6.8811	7.7710
16	Asn (N)	n.d.	n.d.	-7.0065	7.4471
17	Ile (I)	n.d.	n.d.	-6.8133	7.6714
18	Gln (Q)	n.d.	n.d.	-6.8371	7.3236
19	Gly (G)	n.d.	n.d.	-7.1691	7.3630
20	Pro (P)	n.d.	n.d.	-6.8538	7.5590

ΔE (E_{LUMO} - E_{HOMO}), E_{LUMO}, and E_{HOMO} of the optimized molecules were calculated under the level of M06-2X-D3/def2-TZVP. The radical scavenging activity was evaluated using IC_{50}, and the "n.d." means no data.

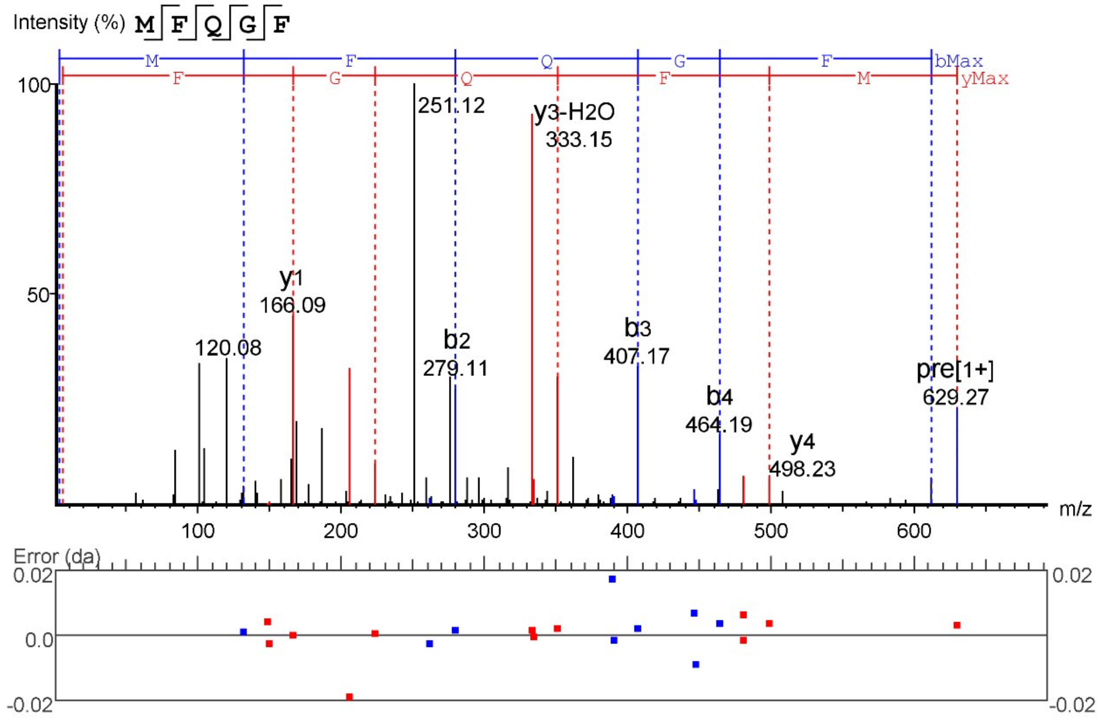

Fig. S9 The MS/MS data of RASP MFQGF.

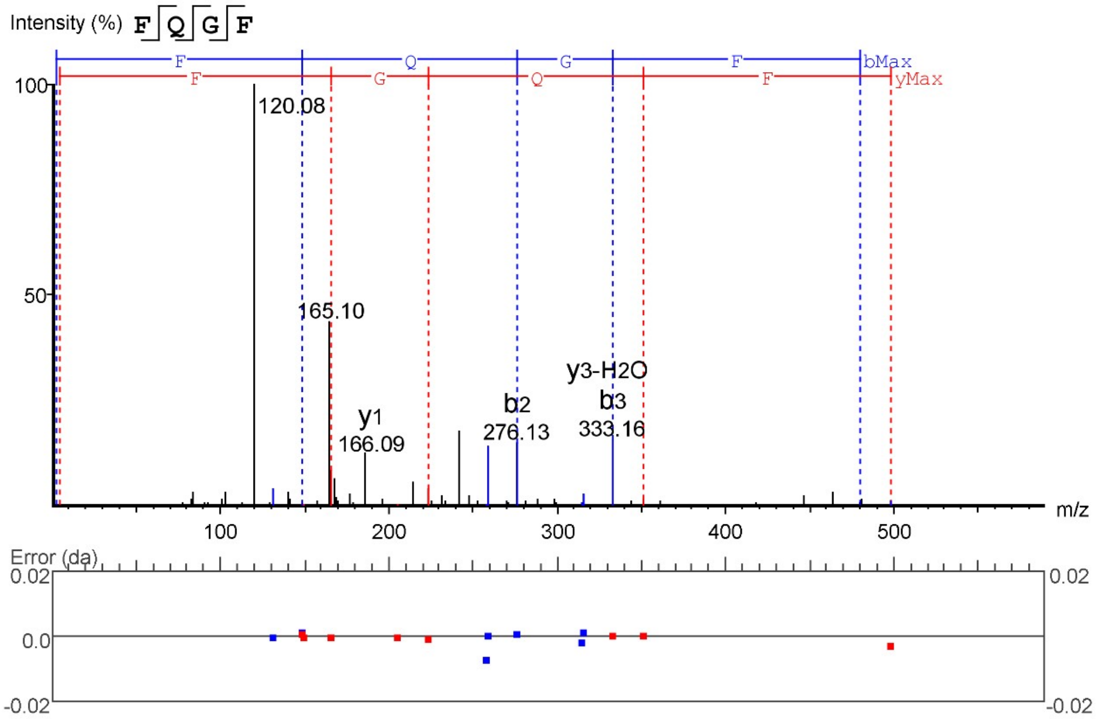

Fig. S10 The MS/MS data of RASP FQGF.

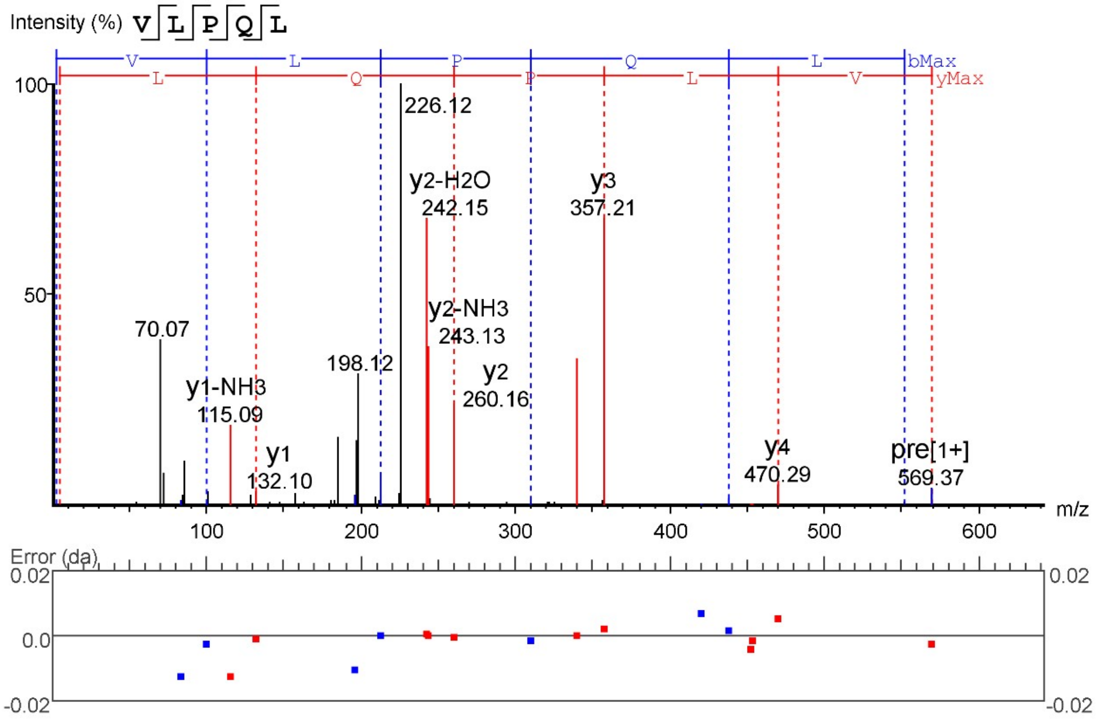

Fig. S11 The MS/MS data of RASP VLPQL.

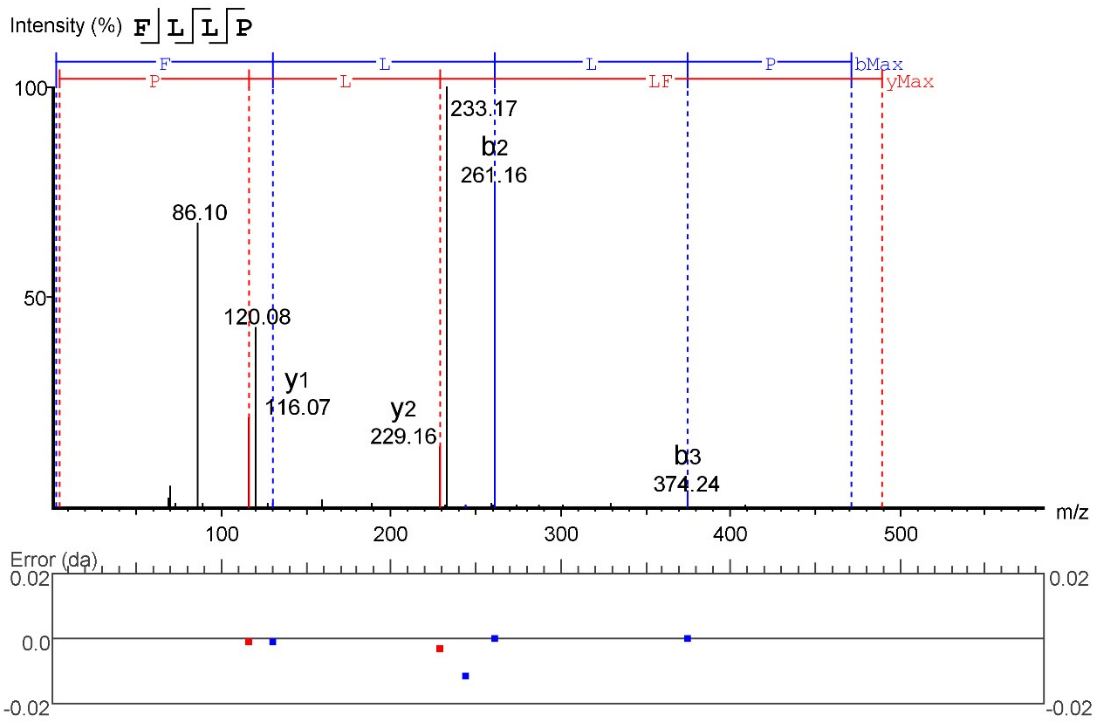

Fig. S12 The MS/MS data of RASP FLLP.

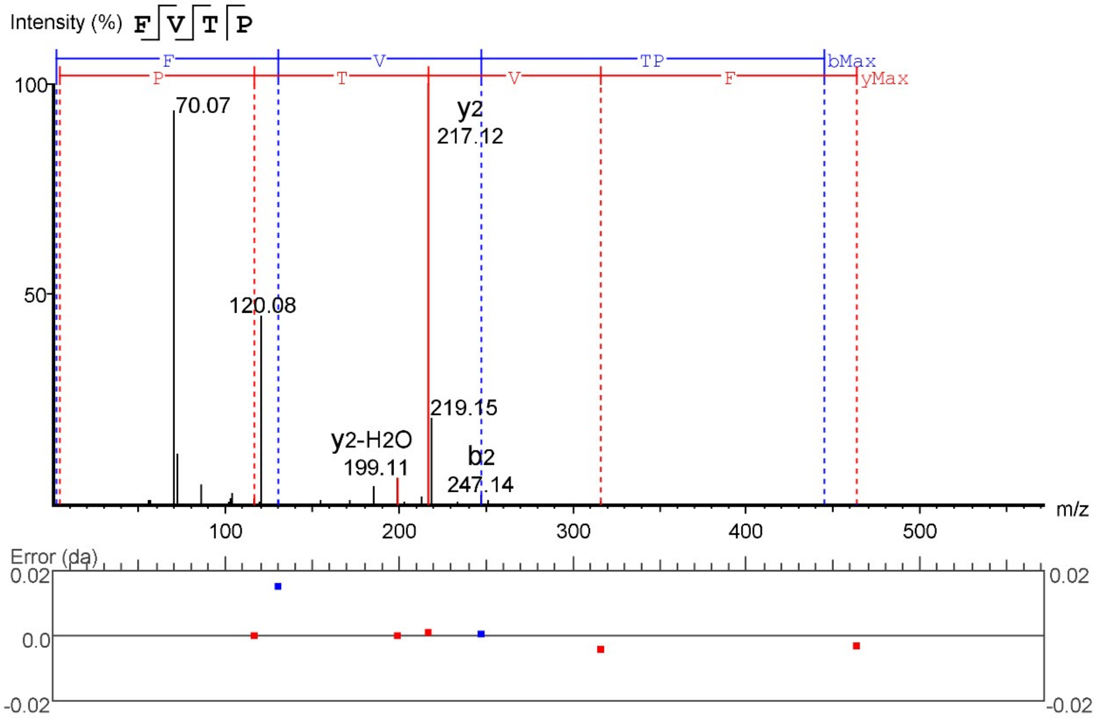

Fig. S13 The MS/MS data of RASP FVTP.

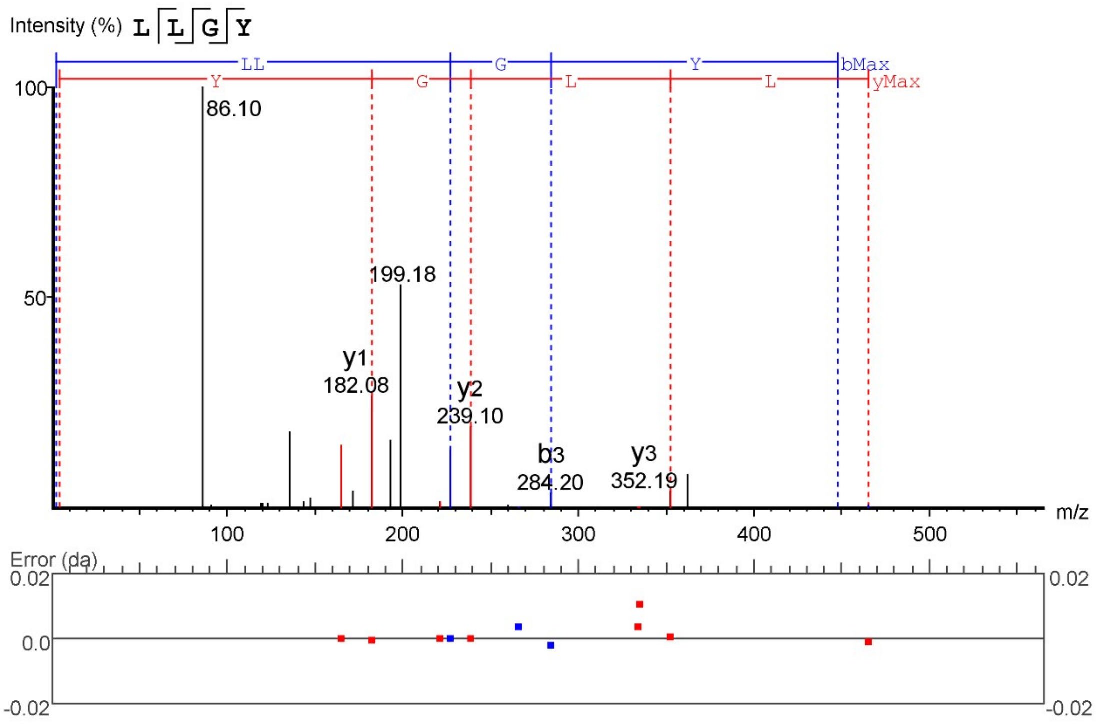

Fig. S14 The MS/MS data of RASP LLGY.

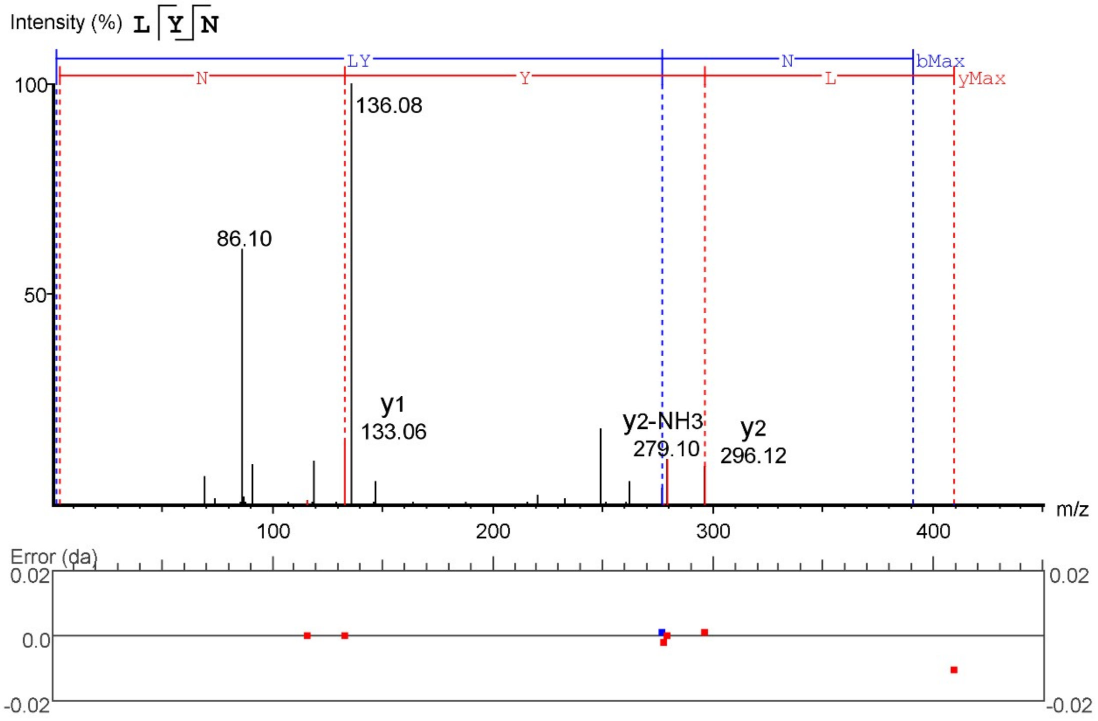

Fig. S15 The MS/MS data of RASP LYN.

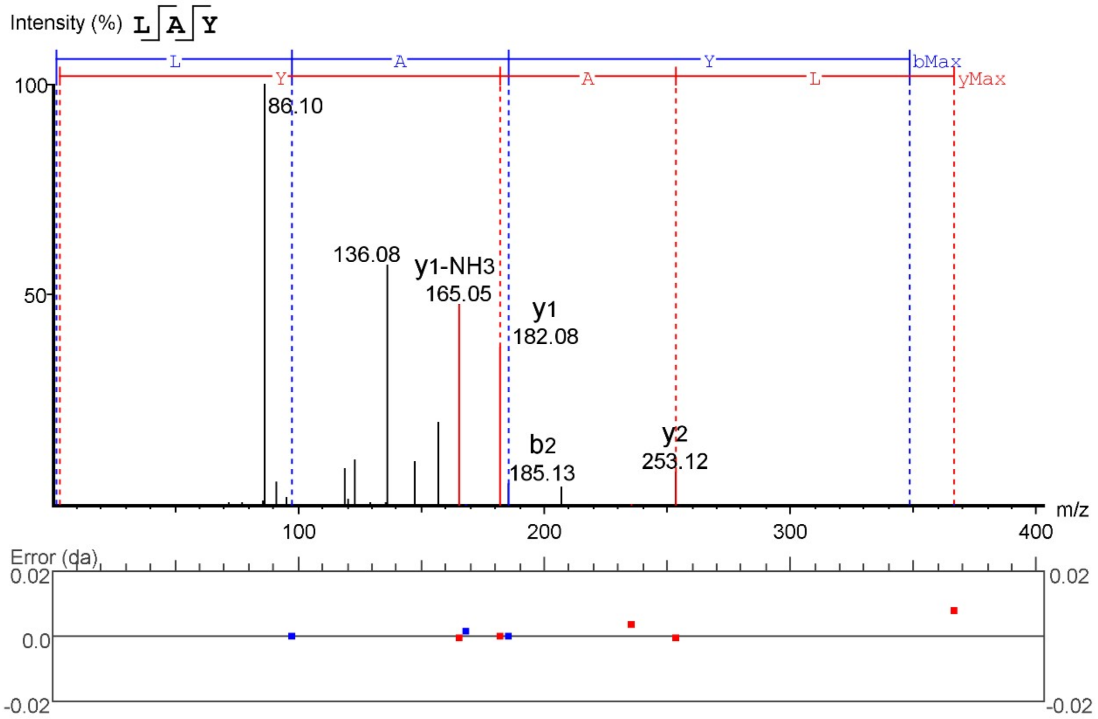

Fig. S16 The MS/MS data of RASP LAY.

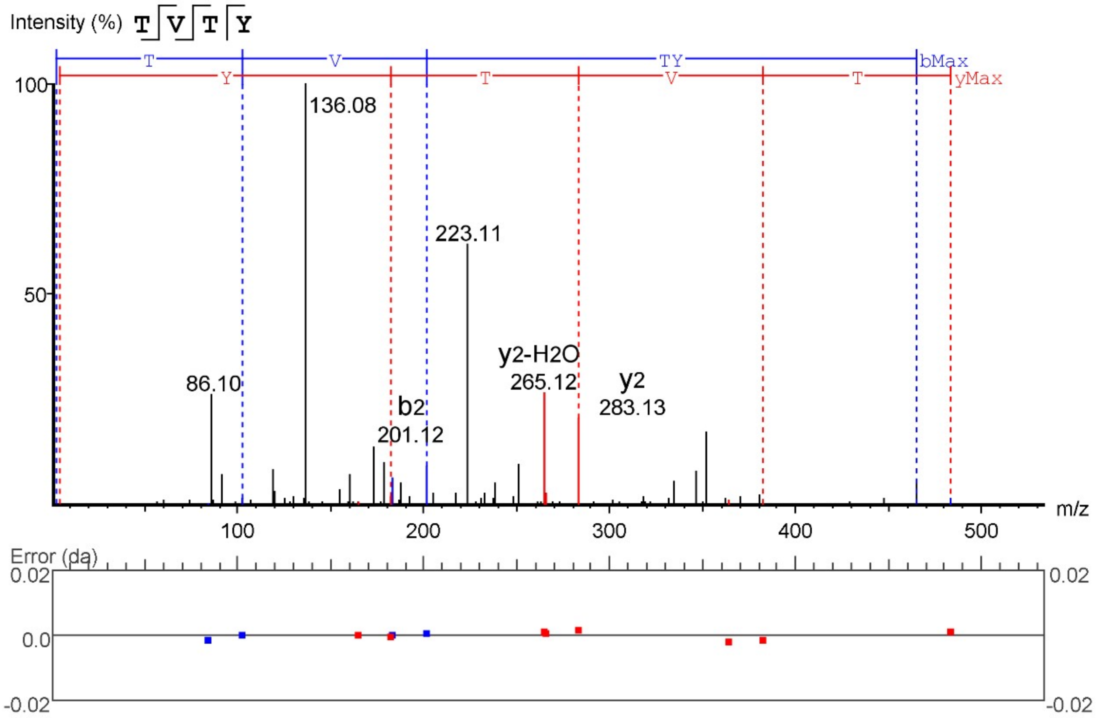

Fig. S17 The MS/MS data of RASP TVTY.

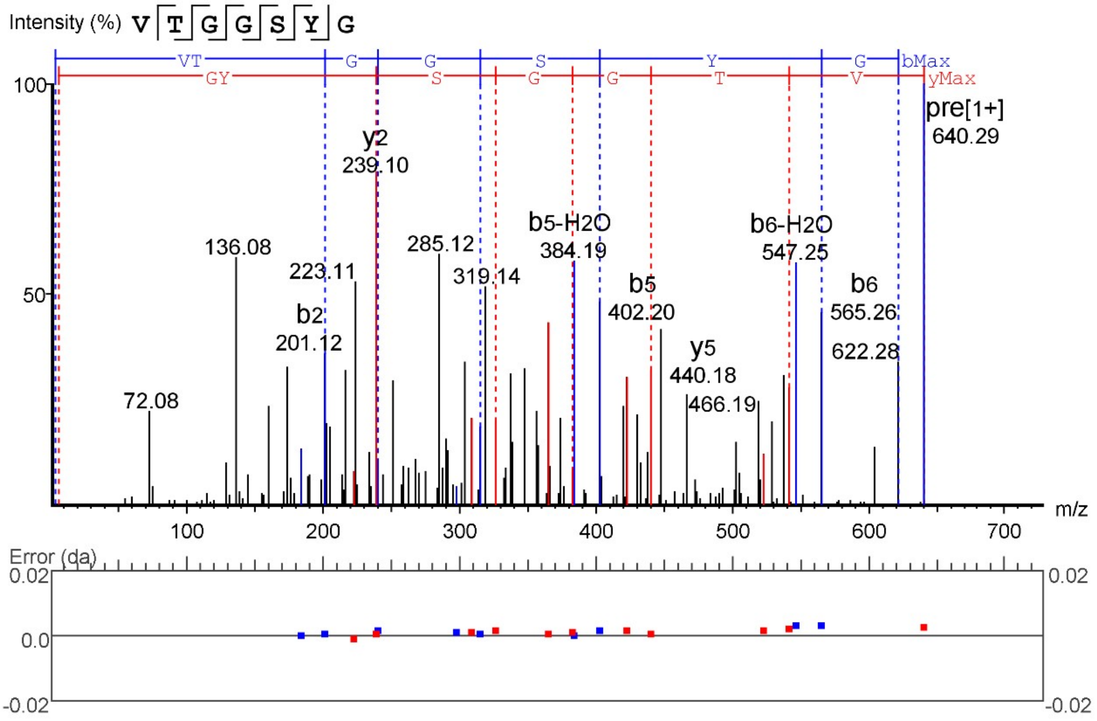

Fig. S18 The MS/MS data of RASP VTGGSYG.

Table S10 *Pearson* correlation analysis between IC_{50}, E_{HOMO}, and ΔE (r, P).

	DPPH	ABTS	E_{HOMO} (eV)	ΔE (eV)
DPPH	1	0.807**	-0.780**	0.731**
ABTS		1	-0.692**	0.661**
E_{HOMO} (eV)			1	-0.981**
ΔE (eV)				1

ΔE (E_{LUMO} - E_{HOMO}), E_{LUMO}, and E_{HOMO} of the optimized molecules were calculated under the level of M06-2X-D3/def2-TZVP. DPPH and ABTS indicated the IC_{50} value of the corresponding radical scavenging assay, $^{**}P < 0.01$.

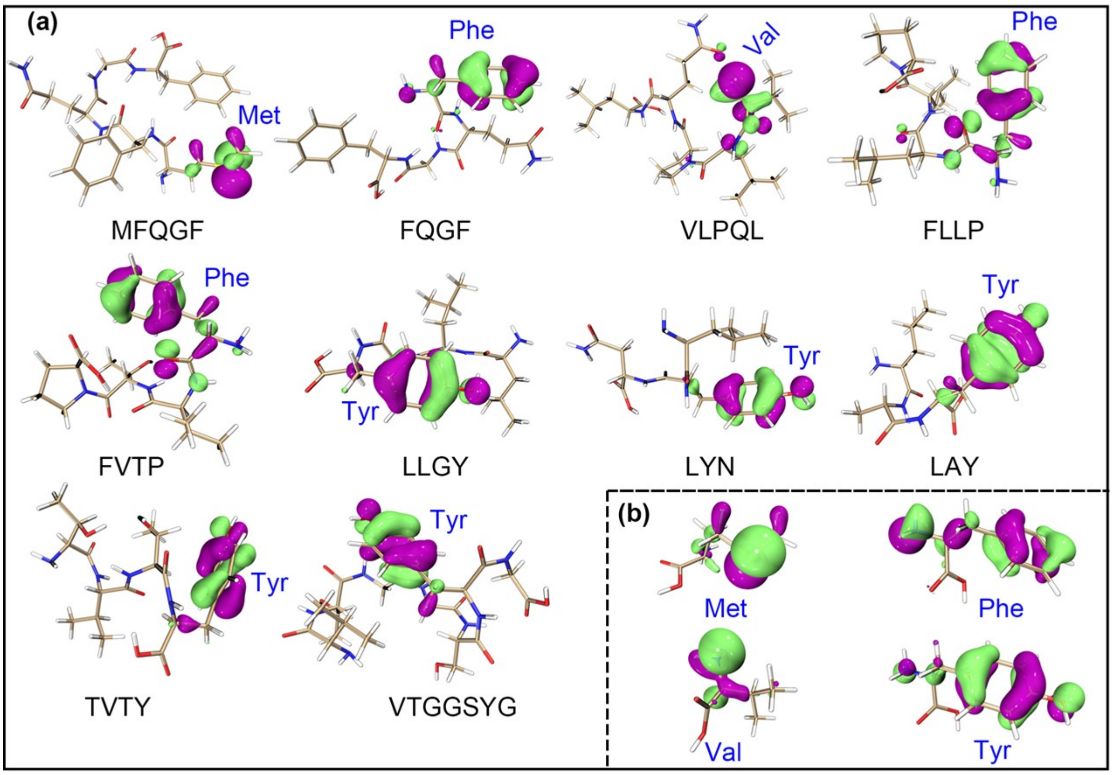

Fig. S19 HOMOs of the 10 RASPs (a) and antioxidant amino acids (b).

In the isosurface map, lime and purple colors correspond to the regions where the spin population was positive and negative, respectively (isovalue = 0.008).

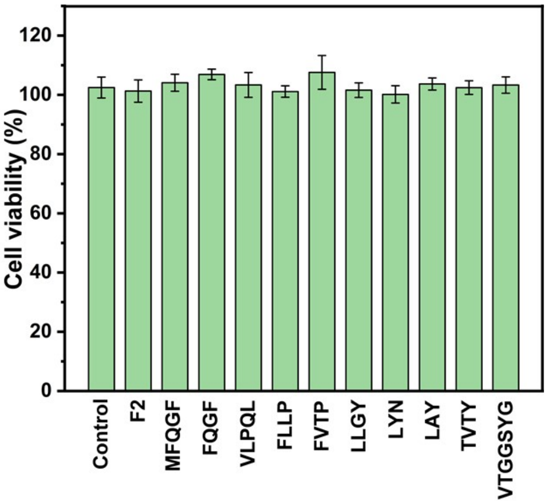

Fig. S20 HT22 cell viability under incubation with F2 fraction (500 μg/mL) or RASPs (1000 μM) for 24 h.

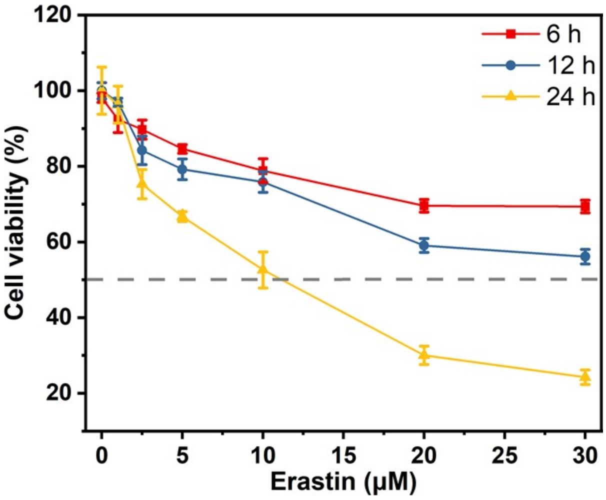

Fig. S21 HT22 cell viability under the incubation of erastin at various concentrations and times.

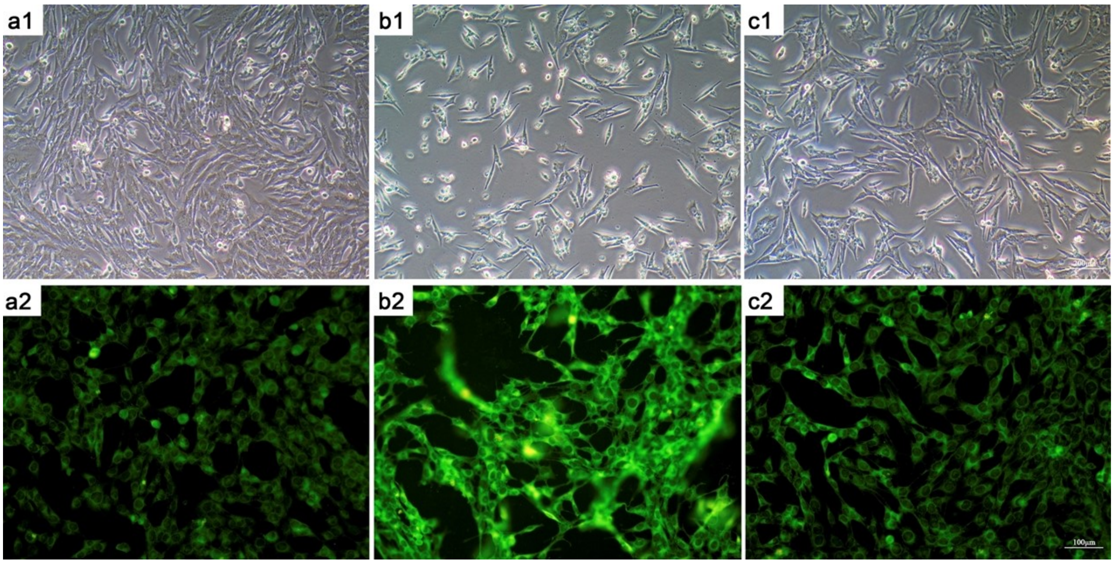

Fig. S22 Effect of erastin on HT22 cell morphology (a1~c1, 100X) and intracellular LPO content (a2~c2, 200X). (a) blank control group; (b) model group (incubated with erastin); (c) control group (incubated with erastin and Fer-1).

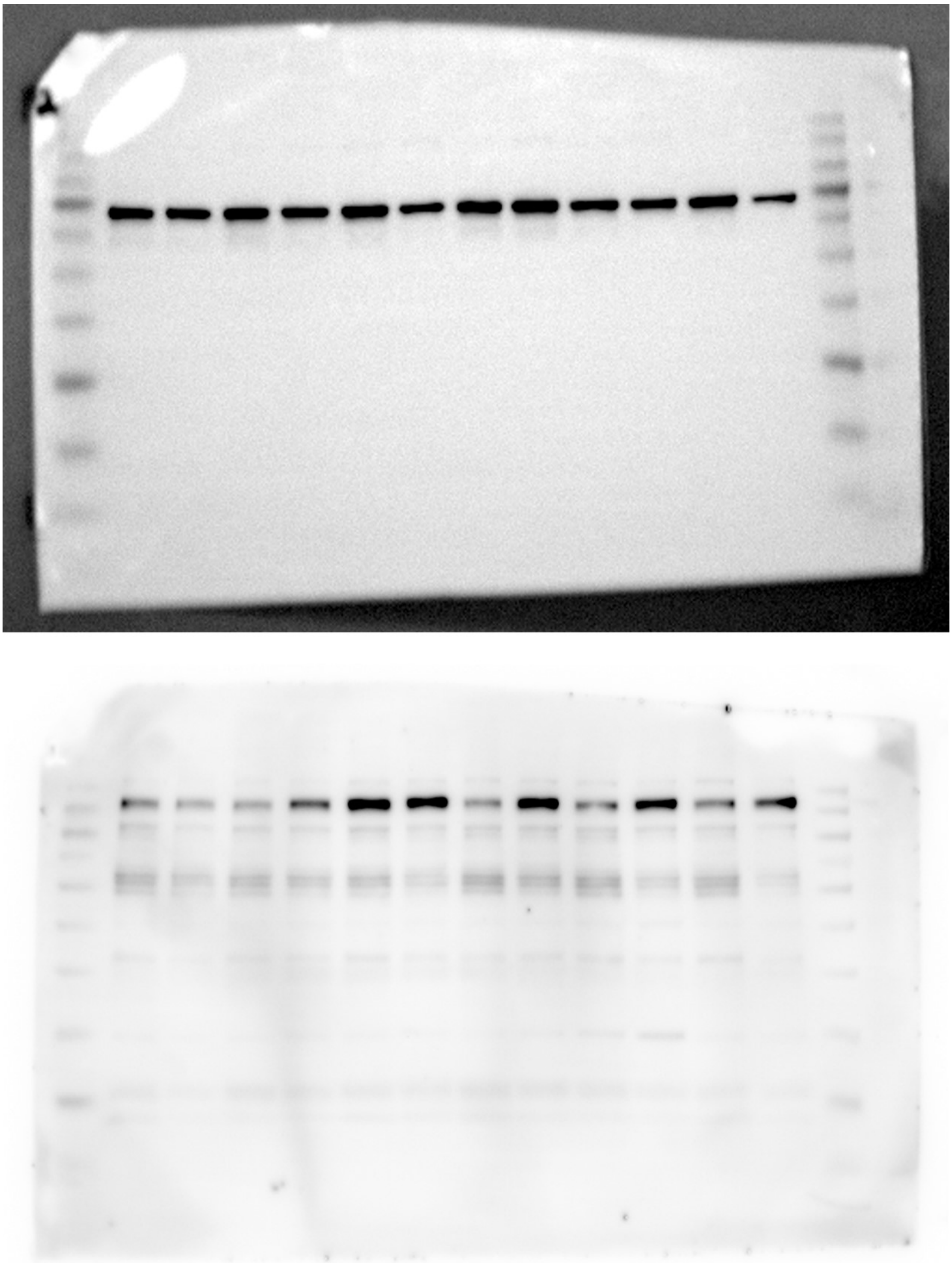

Fig. S23 Raw data of Western blots.

The top panel showed the expression of Lamin B and the bottom panel showed the expression of Nrf2. From left to right, they were groups of Control, control + ML385, model, model + ML385, MFQGF, MFQGF + ML385, LLGY, LLGY + ML385, F2, F2 + ML385, Fer-1, and Fer-1 + ML385. Since the inhibitory effect of Nrf2 by ML385 was not within the scope of this study, their data were not presented in the main text.

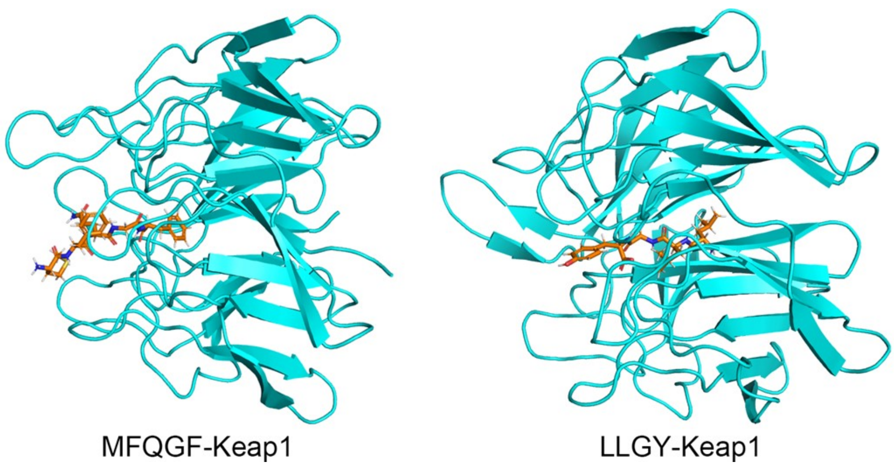

Fig. S24 3D conformation of MFQGF-Keap1 and LLGY-Keap1 obtained from MD simulation.

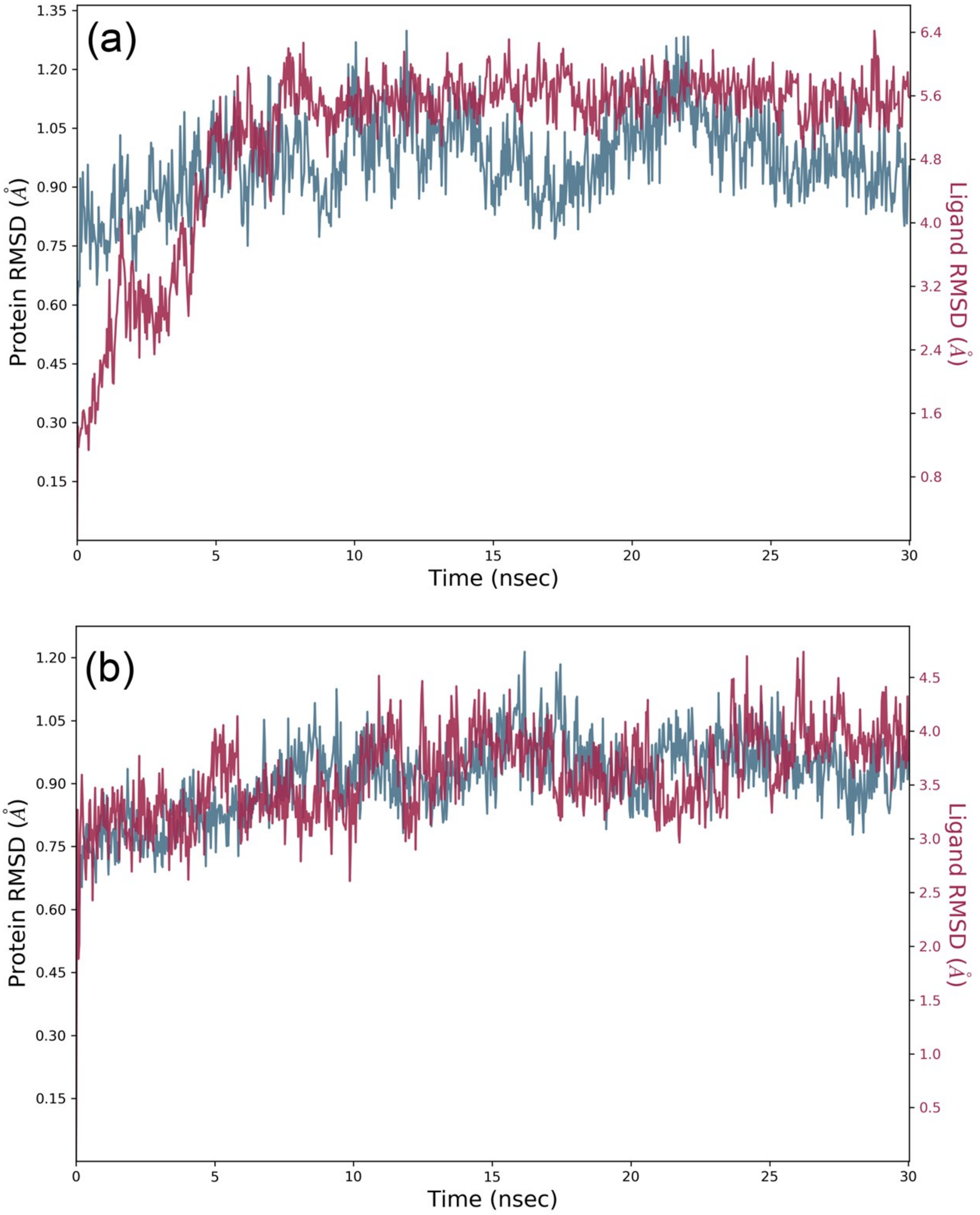

Fig. S25 RMSD of MFQGF-Keap1 (a) and LLGY-Keap1 (b) derived from MD simulation.

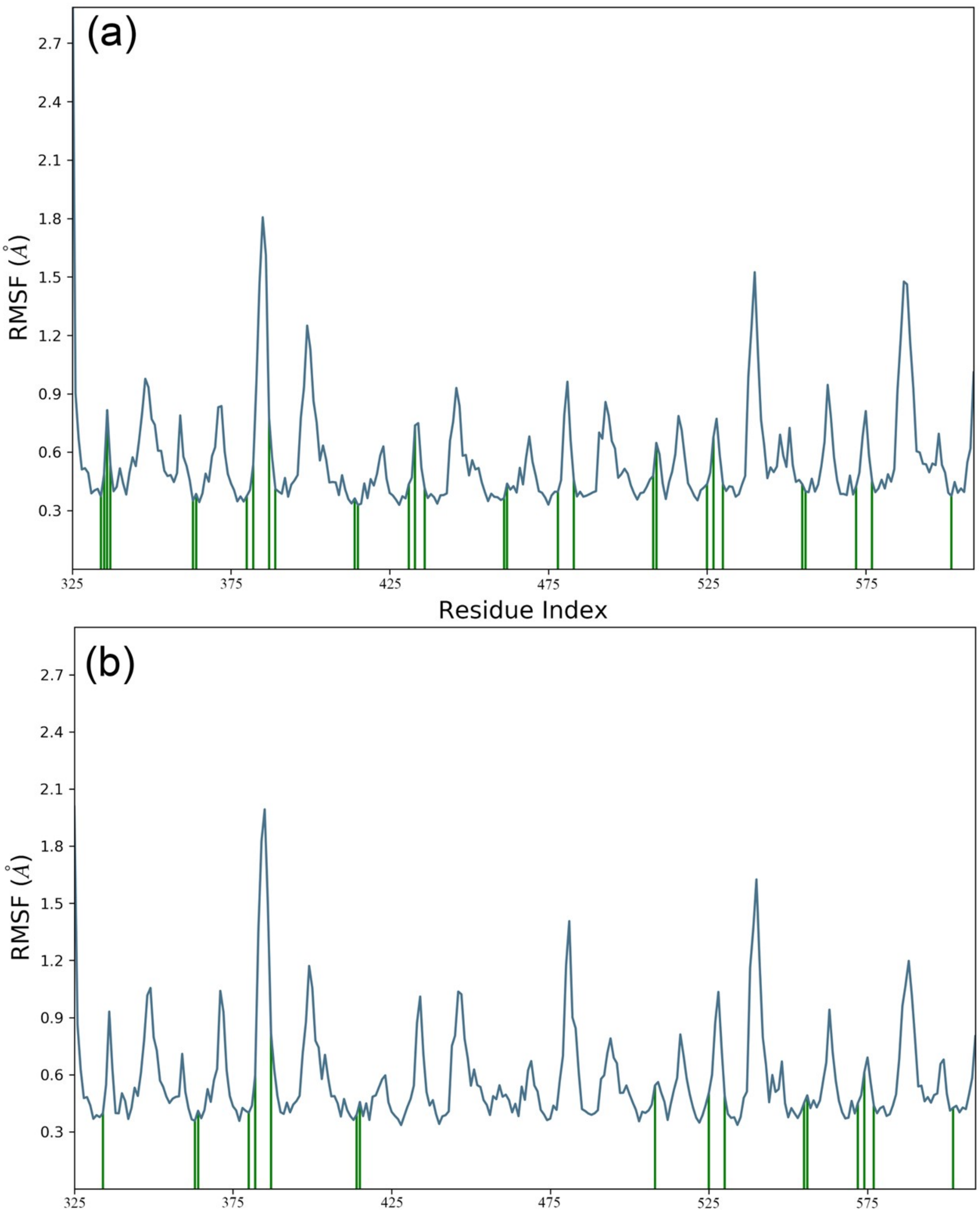

Fig. S26 RMSF of MFQGF-Keap1 (a) and LLGY-Keap1 (b) derived from MD simulation.

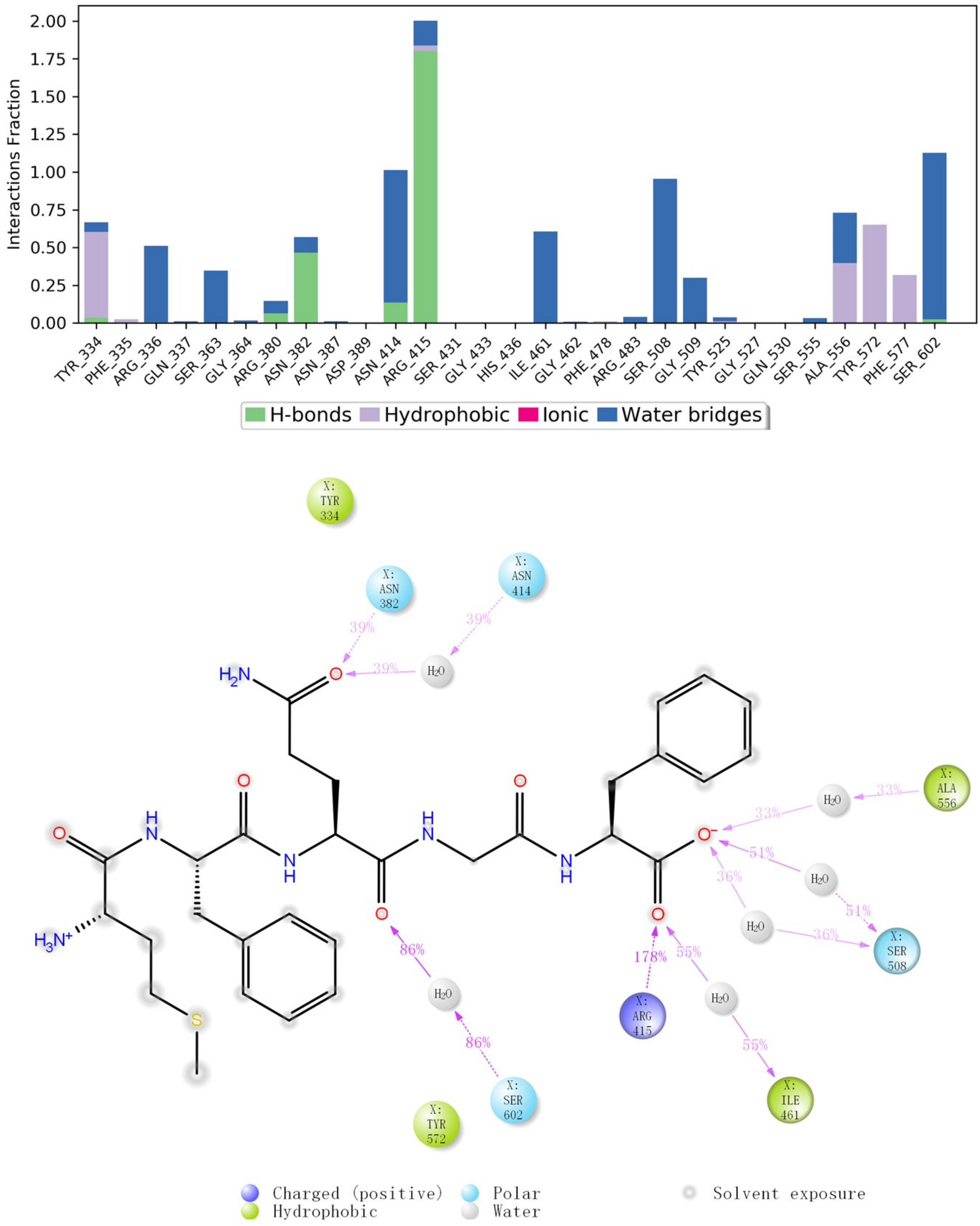

Fig. S27 Interaction mode of the MFQGF-Keap1.

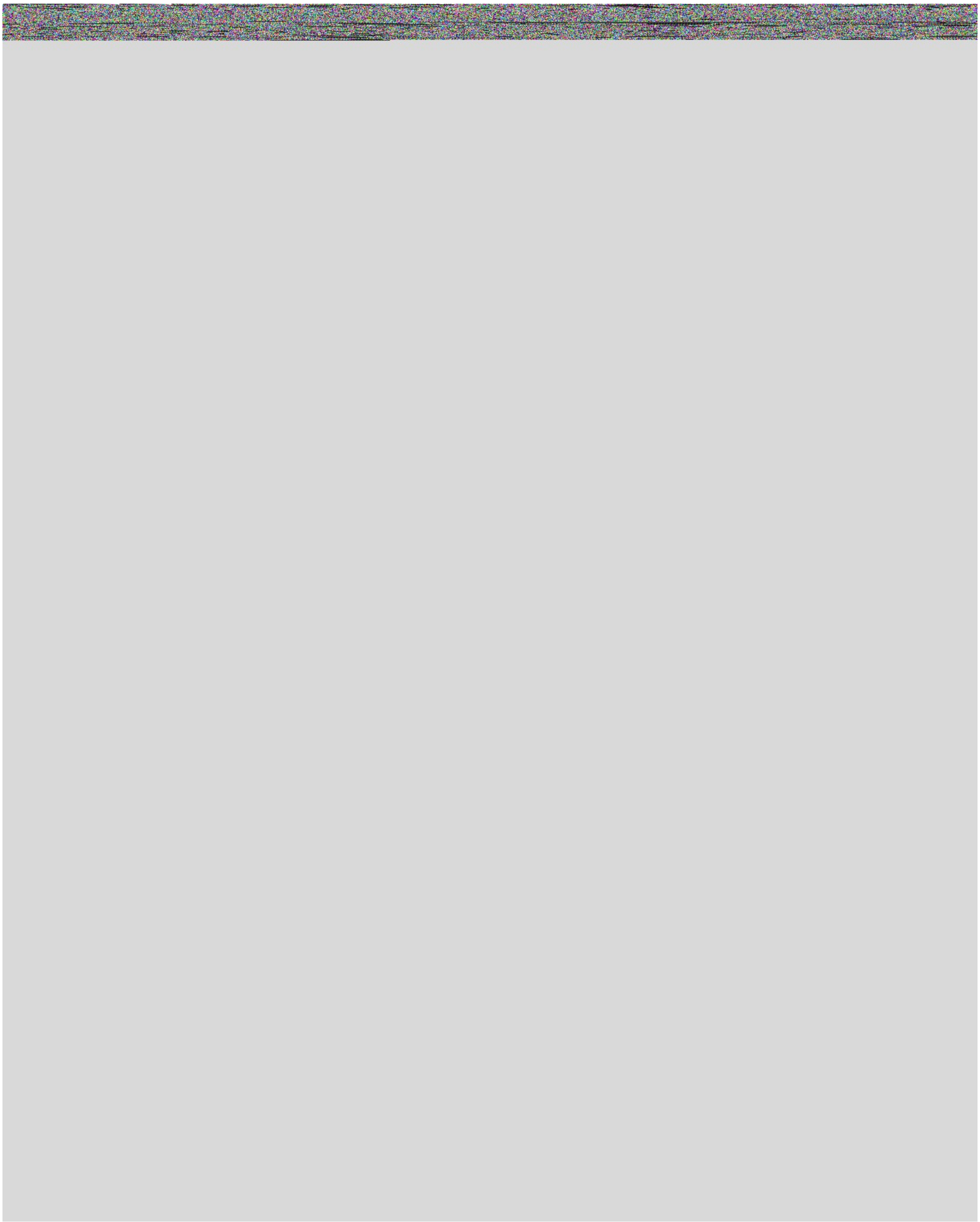

Fig. S28 Interaction mode of the LLGY-Keap1.

Table S11 Binding free energies and energy components predicted by MM/GBSA (kcal/mol).

System	Keap1-Nrf2	Keap1-MFQGF	Keap1-LLGY
ΔE_{vdw}	-53.56 ± 5.31	-44.69 ± 3.82	-20.09 ± 4.76
ΔE_{elec}	-335.71 ± 38.03	-80.75 ± 14.05	-133.06 ± 13.30
ΔG_{GB}	334.63 ± 37.14	105.60 ± 11.67	135.46 ± 12.78
ΔG_{SA}	-8.89 ± 0.39	-6.23 ± 0.35	-3.61 ± 0.67
ΔG_{bind}	-63.54 ± 5.02	-26.07 ± 3.91	-21.31 ± 5.45

ΔE_{vdW}: van der Waals energy.

ΔE_{elec}: electrostatic energy.

ΔG_{GB}: electrostatic contribution to solvation.

ΔG_{SA}: non-polar contribution to solvation.

ΔG_{bind}: binding free energy.
